# Type 1 diabetes, glycemic traits, and risk of dental caries: a Mendelian randomization study

**DOI:** 10.3389/fgene.2023.1230113

**Published:** 2023-10-10

**Authors:** Li Tan, Meng-Mei Zhong, Ya-Qiong Zhao, Jie Zhao, Marie Aimee Dusenge, Yao Feng, Qin Ye, Jing Hu, Ze-Yue Ou-Yang, Ning-Xin Chen, Xiao-Lin Su, Qian Zhang, Qiong Liu, Hui Yuan, Min-Yuan Wang, Yun-Zhi Feng, Yue Guo

**Affiliations:** Department of Stomatology, The Second Xiangya Hospital, Central South University, Changsha, Hunan, China

**Keywords:** Mendelian randomization analysis, diabetes mellitus, type 1, glycemic traits, dental caries, multidisciplinary intervention

## Abstract

**Background:** Regarding past epidemiological studies, there has been disagreement over whether type 1 diabetes (T1DM) is one of the risk factors for dental caries. The purpose of this study was to determine the causative links between genetic susceptibility to T1DM, glycemic traits, and the risk of dental caries using Mendelian randomization (MR) approaches.

**Methods:** Summary-level data were collected on genome-wide association studies (GWAS) of T1DM, fasting glucose (FG), glycated hemoglobin (HbA1c), fasting insulin (FI), and dental caries. MR was performed using the inverse-variance weighting (IVW) method, and sensitivity analyses were conducted using the MR-Egger method, weighted median, weighted mode, replication cohort, and multivariable MR conditioning on potential mediators.

**Results:** The risk of dental caries increased as a result of genetic susceptibility to T1DM [odds ratio (OR) = 1.044; 95% confidence interval (CI) = 1.015–1.074; *p* = 0.003], with consistent findings in the replication cohort. The relationship between T1DM and dental caries was stable when adjusted for BMI, smoking, alcohol intake, and type 2 diabetes (T2DM) in multivariable MR. However, no significant correlations between the risk of dental caries and FG, HbA1c, or FI were found.

**Conclusion:** These results indicate that T1DM has causal involvement in the genesis of dental caries. Therefore, periodic reinforcement of oral hygiene instructions must be added to the management and early multidisciplinary intervention of T1DM patients, especially among adolescents and teenagers, who are more susceptible to T1DM.

## Background

Type 1 diabetes (T1DM) is a dangerous and prevalent progressive disease. The condition is caused by autoimmune inflammation that damages the beta cells in the pancreatic islets of Langerhans, which produce the hormone insulin (Eizirik et al., 2009). This disease, which affects children and adolescents more frequently than adults, has been found to share several pathogenic genes with other illnesses, such as hypothyroidism and non-alcoholic fatty liver disease (Ferizi et al., 2022; Zhao et al., 2022; Liu et al., 2023). Better management of T1DM patients has become a vital issue due to the uniqueness of its predisposing age and the complexity of the disease. Therefore, exploring diseases that may be related to T1DM is conducive to their management and early multidisciplinary intervention (Zafra-Tanaka et al., 2022).

A common chronic infectious disease that affects the hard tissues of teeth is dental caries (Selwitz et al., 2007). Dental caries is a very common symptom that ranks 11th among all diseases worldwide in terms of prevalence, according to a recent *Lancet* report (GBD, 2015). Dental caries develop and arise as a result of environmental and genetic variables that are not fully understood (Opal et al., 2015). Furthermore, dental caries and its associated complications can lead to or exacerbate systemic disorders that greatly reduce human quality of life (Sabharwal et al., 2021).

Recent studies have shown that T1DM is associated with various oral complications (Bimstein et al., 2019). However, there is no consensus regarding the association between T1DM and dental caries (Sampaio et al., 2011; Novotna et al., 2015). The common risk factors for dental caries include oral cariogenic bacteria, intake of fermentable carbohydrates as a substrate for cariogenic bacteria, and sufficient time for caries formation. The protective factors against dental caries include saliva, oral hygiene, and fluorides (Pitts et al., 2017). The present study suggests that some factors increase the risk of distal caries in T1DM patients, while others reduce the risk. Some studies have shown that the level of cariogenic bacteria, particularly *Lactobacillus*, is higher in T1DM patients, which leads to a high risk of dental caries (Ferizi et al., 2018). Other studies have, however, revealed that the oral hygiene of T1DM patients seems to be slightly better than that of healthy individuals, which may lead to a reduction in the risk of dental caries (Manjushree et al., 2022). Observational research has additionally demonstrated inconsistencies in the linkage between T1DM and dental caries. A meta-analysis involving 538 individuals found a higher incidence of dental caries in T1DM patients than in healthy controls (Wang et al., 2019). However, in a 2-year cohort study conducted by Siudikiene et al. (2008), there were no significant differences in the incidence of dental caries between T1DM patients and healthy control groups. The reason for this dispute may be that nearly all of the aforementioned findings about the link between T1DM and dental caries are based on traditional observational studies, which may have some intrinsic flaws, including the potential of reverse causality and residual confounding (Boorsma et al., 2019). These studies may not have controlled for variables such as lifestyle and eating habits when selecting their observation objects, which may have affected the data on the incidence of dental caries. This could have resulted in residual confounding.

To address these limitations and to better understand the causal relationship between T1DM and dental caries, we employed the Mendelian randomization (MR) analysis to determine whether T1DM would increase the risk of dental caries. Mendel’s second law is utilized by the MR analysis, which views gene variants as instrumental variables. It is possible to circumvent the biases associated with traditional research methods such as observational studies (e.g., reverse causality and residual confounding) by randomly allocating genotypes before conception, which simulates natural, randomized, and controlled study circumstances (Skrivank et al., 2021).

In this work, we obtained single-nucleotide polymorphisms (SNPs) of T1DM and glycemic traits such as fasting glucose (FG), glycated hemoglobin (HbA1c), and fasting insulin (FI) for instrumental variables in the open-access genome-wide association studies (GWAS) database. FG and HbA1c are glycemic traits used to diagnose diabetes. In addition, HbA1c is the most commonly used biomarker to monitor glucose control in patients with diabetes (WHO, 2011). FI reflects the severity of islet β-cell dysfunction (Heise et al., 2022). Collectively, all three glycemic traits are useful to better understand T1DM pathophysiology and the outcome of dental caries. Furthermore, we also extracted the outcome of dental caries in this database. Finally, with the help of the R package “TwoSampleMR,” inverse-variance weighting (IVW) and sensitivity analyses were carried out to determine how T1DM affects dental caries.

## Materials and methods

The STROBE-MR checklist of recommended items to address reports of MR studies was followed in our study (Skrivank et al., 2021) (Supplementary Table S1).

### Study design and data sources

**FIGURE 1:**
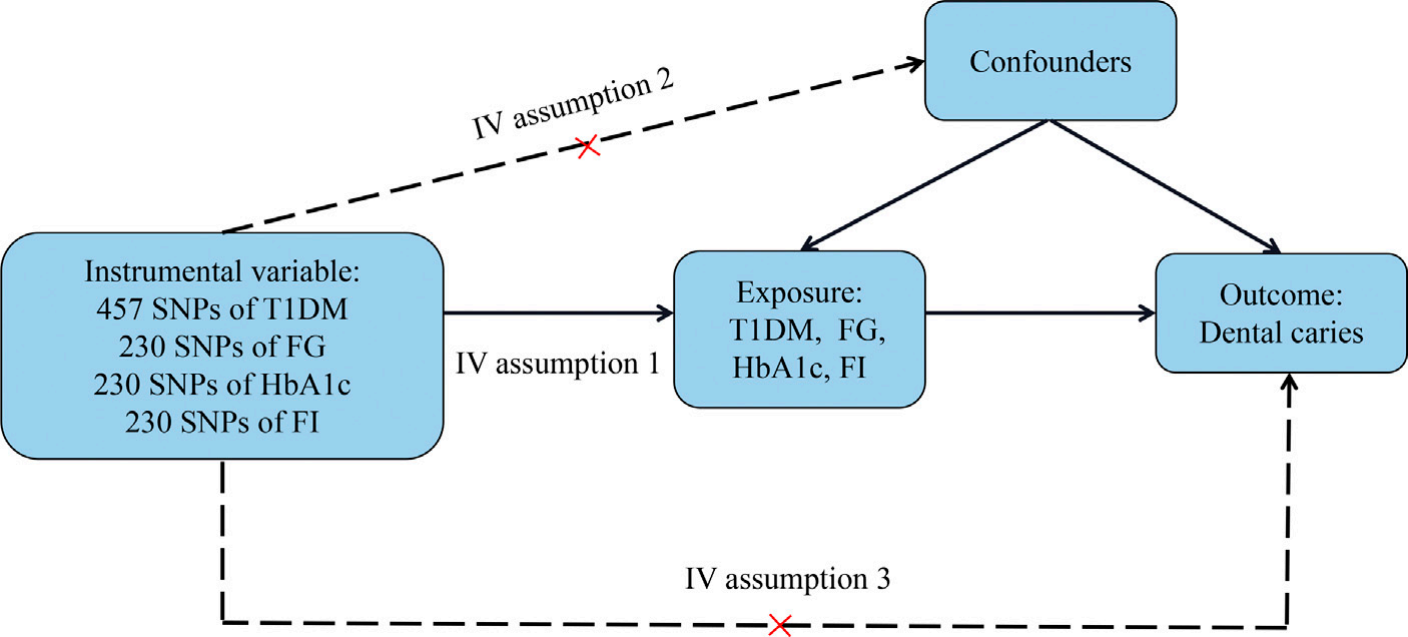
Causal diagram of Mendelian randomization in whether T1DM or glycemic traits could affect dental caries. IV assumption: 1) the exposure and IV are linked; this is often referred to as the relevancy assumption; 2) it is unaffected by confounding factors that can be measured or not, also known as the independence assumption; and 3) it can only influence the outcome through the exposure, also known as the exclusion restriction assumption.

Our design is shown in Figure 1 and adheres to the three assumptions of Mendel’s randomized design principle (Skrivank et al., 2021). As shown in Figure 1, a valid instrumental variable (IV) must satisfy three assumptions: 1) the exposure and IV are linked, which is often referred to as the relevancy assumption; 2) it is unaffected by confounding factors that can be measured or not, also known as the independence assumption; and 3) it can only influence the outcome through exposure, also known as the exclusion restriction assumption. Our IV selection is described below and meets the three aforementioned assumptions. Furthermore, we chose GWAS summary data for the exposure and outcome from different study cohorts. We also selected GWAS summary data of the outcome from different consortia as replication cohorts for further research when designing the MR study to avoid a large overlap of samples between the exposure and outcome. This is because a significant sample overlap between the exposure and outcome consortia could skew two-sample MR estimates in favor of the confounded connection between exposure and outcome (Burgess et al., 2016).

In terms of the selection of GWAS summary data for T1DM, we referred to the design of recent MR studies on T1DM [for example, the MR study by Yazdanpanah et al. (2022) published in *Diabetes Care* in 2022] (Manousaki et al., 2021), all of which had selected the large T1DM GWAS meta-analysis from Forgetta et al. (2020). The included T1DM GWAS data had a total sample size of 24,840 individuals of European ancestry (9,266 T1DM cases and 15,574 controls from 12 European cohorts) (for a detailed description of the T1DM GWAS data, see the study by Forgetta et al., 2020). We obtained exposure data on FG, HbA1c, and FI from the Meta-Analyses of Glucose and Insulin-related traits Consortium (MAGIC) GWAS, which included 336,639, 146,806, and 151,013 European individuals, respectively. The body mass index (BMI), study-specific covariates, and principal components were regressed on these glycemic traits with the aim of adjusting for potential confounding variables to better understand the relationship between the glycemic trait and genome-wide significant variants identified in these GWAS (all information on these GWAS data can be found in the study of Chen et al., 2021).

Then, to satisfy the relevance assumption, all the SNPs of exposure that met the genome-wide significant criteria (*p* < 5 × 10^−8^) were chosen for this investigation. Additionally, we determined the physical distance among SNPs >10,000 kb by configuring the clump data function in the TwoSampleMR package, and the *R* ^2^ of the linkage disequilibrium (LD) relationship between genes <0.001 was constructed, avoiding potential bias brought on by the LD relationship between the SNPs.

The GWAS summary statistics data for dental caries (4,170 cases and 195,395 controls of European ancestry) were obtained from the FinnGen biobank, a large-scale project in Finland with over 500,000 participants that employs a unique study design to avoid sample overlap and reduce bias. FinnGen offers comprehensive genetic and phenotypic data that can be used to conduct rigorous Mendelian randomization studies investigating causal relationships between risk factors and health outcomes (Yarmolinsky et al., 2022; Kurki et al., 2023). The diagnosed cases of dental caries (K02) were recorded with the International Classification of Diseases (ICD-10). The ICD-10 code for dental caries (ICD-10-K02) is available at https://www.who.int/publications/m/item/international-statistical-classification-of-diseases-and-related-health-problems---volume-2, as described in the endpoints table—DF11 on the website (https://www.finngen.fi/en/researchers/clinical-endpoints). The results of these GWAS data are summarized by *p*-values, SE, and values Supplementary Table S8. The analyses that produced the public information utilized in this study were restricted to demographic data from the European population, and Supplementary Table S2 provides a summary of these topics.

To further verify the relevance assumption, we computed the F-statistic of T1DM and glycemic traits (Supplementary Tables S3–S6) by all SNPs. The 39 SNPs for T1DM had a minimum F-statistic of 30.54, and the F-statistic of glycemic traits (66 SNPs for FG, 73 SNPs for HbA1c, and 38 SNPs for FI) were 24.52, 25.00, and 22.44, respectively. All these values exceeded the commonly accepted threshold of F > 10, which indicates that bias in instrumental variable analysis is avoided and the results are not misleading (Burgess et al., 2011). Furthermore, the proportion of variation explained by all the variants that we have considered to be instrumental variables for the exposures ranged from 1.3% (for FI) to 17.7% (for T1DM) (Supplementary Tables S3–S6).

### Mendelian randomization analysis

In this work, the primary analysis method was inverse-variance weighting (IVW). Additionally, the MR-Egger, weighted mode, and median weighted methods were employed as Supplementary Methods. Under the assumption that all instrumental variables were effective, the IVW principle used each instrumental variable’s reciprocal variance as a weight in weighted computations. As a result, all instrumental variable effect values were weighted according to the variance, where estimates with larger SE were weighted less in the IVW estimate. The TwoSampleMR in the R package (V.4.1.2) was used to carry out the abovementioned analysis. We did not use proxy SNPs when our genetic variants of interest were not available in the outcome GWAS summary statistics.

### Sensitivity analysis

Initially, using the Cochran’s Q test and I^2^ statistic, we examined the heterogeneity of the SNPs of all the exposures. Then, to satisfy the exclusion restriction assumption, we used the MR-PRESSO package to detect horizontal pleiotropy (Verbanck et al., 2018). Next, the horizontal pleiotropy of the instrumental variables was also determined using the Egger intercept method (Bowden et al., 2015). If the intercept term’s *p*-value in the regression equation was greater than 0.05, no evidence of horizontal pleiotropy was considered. Similarly, we carried out a leave-one-out analysis using the TwoSampleMR package to confirm the stability of the analysis. The TwoSampleMR package also produced the forest plot and funnel plot. Furthermore, we satisfied the independence assumption by scanning the PhenoScanner database, which was the method used to find SNPs associated with potential confounders (Kamat et al., 2019). Then, we selected and excluded these SNPs (e.g., obesity, smoking, alcohol consumption, etc.) according to previously reported studies. All the excluded SNPs are summarized in Supplementary Table S7. Finally, we also conducted a multivariable MR analysis to determine potential mediators or confounders, which considers the association of variants with multiple exposures (Burgess and Thompson, 2015). We employed the multivariable MR approach using the IVW method to investigate the association between T1DM and dental caries, while adjusting for the effects of variants linked to T2DM (type 2 diabetes) (Bonàs-Guarch et al., 2018), smoking (Liu et al., 2019), alcohol intake (Liu et al., 2019), and BMI (Wood et al., 2016). Specifically, we gathered all SNPs related to T1DM or potential mediators or confounders that were significant across the genome and clumped them with pairwise LD *r* ^2^ < 0.001, determined by the lowest *p*-value for their association with any trait, when choosing instruments for multivariable MR analyses.

### Multiple testing

Because of a great deal of statistical tests performed using univariable MR, we employed the Bonferroni correction for multiple testing (Chen et al., 2017). We assessed four exposures, namely, T1DM, FG, HbA1c, and FI, which were divided into four clusters. Consequently, we corrected the significance level from *p* = 0.05 to *p* = 0.0125 to account for multiple testing. However, it is important to note that this correction method has limitations and may lead to some true positive results being rejected (Armstrong, 2014). To supplement this approach, we also used the Benjamini–Hochberg correction to control the false discovery rate (FDR) by considering the statistical significance when the corrected *p*-value (q-value) was less than 0.5 (Benjamini and Hochberg, 1995) (Table 1).

**TABLE 1:** Multiple testing for results of univariable MR using the Benjamini–Hochberg correction (FDR <0.05) or Bonferroni correction (*p* < 0.0125).

Correlation: Dental caries and	*p*-value	Rank	*p* (m/i)	Benjamini–Hochberg corrected *p*-value significant? (FDR <0.05)	Bonferroni corrected *p*-value significant? (*p* < 0.0125)
			Benjamini–Hochberg		
			Corrected *p*-value		
T1DM (IVW)	0.003	2	0.006	Yes	Yes
FG (IVW)	0.597	3	NA	No	No
HbA1c (IVW)	0.623	4	NA	No	No
FI (IVW)	0.002	1	0.008	Yes	Yes
T1DM (MR-Egger)	0.014	2	0.028	Yes	No
FG (MR-Egger)	0.953	4	NA	No	No
HbA1c (MR-Egger)	0.005	1	0.020	Yes	Yes
FI (MR-Egger)	0.940	3	NA	No	No
T1DM (weighted median)	0.009	1	0.036	Yes	Yes
FG (weighted median)	0.781	3	NA	No	No
HbA1c (weighted median)	0.960	4	NA	No	No
FI (weighted median)	0.122	2	NA	No	No
T1DM (weighted mode)	0.004	1	0.016	Yes	Yes
FG (weighted mode)	0.796	4	NA	No	No
HbA1c (weighted mode)	0.644	3	NA	No	No
FI (weighted mode)	0.472	2	NA	No	No

i, the individual *p*-value’s rank; m, total number of tests; FDR, false discovery rate; NA, not applicable.

### Replication cohort

In MR, the term “replication cohort” refers to a different data set that is used to confirm the initial research findings. Recently, some MR studies have considered adding it to reduce the possibility of false positives, enhance the statistical power of the study, and provide more reliable evidence for causal inference.

Here, we used a replication cohort to investigate dental caries, which originated from a GWAS meta-analysis data set that combines evidence from two sources (Shungin et al., 2019): the Gene–Lifestyle Interactions in Dental Endpoints (GLIDE) consortium, an exceptional collection of epidemiological cohorts with in-depth information on clinical endpoints of dental diseases, and the UK Biobank (UKB), a sizable data set containing self-reported oral health information. The GWAS meta-analysis data set selected decayed, missing, and filled tooth surfaces (DMFS) and dentures as the pair of clinical and self-reported traits with the greatest shared heritability, representing the progression of dental caries. The data set combined single-variant association statistics from the GLIDE and UKB data sets using a z-score genome-wide meta-analysis weighted by effective sample size. The principal analyses combined DMFS (*n* = 26,792 from nine studies) and dentures (n_cases = 77,714; n_controls = 383,317) in European individuals. Additionally, it is important to note that the binary variable (dentures) in this meta-analysis data set was transformed into a continuous variable (log odds ratio) before being combined with the results of other independent studies (DMFS). This transformation typically requires specific statistical methods to ensure the accuracy and comparability of the results. In this study, we adopted the “z-score genome-wide meta-analysis weighted” method to integrate the results of independent studies with different phenotypes.

## Results

### Genetic association between T1DM and dental caries

**FIGURE 2:**
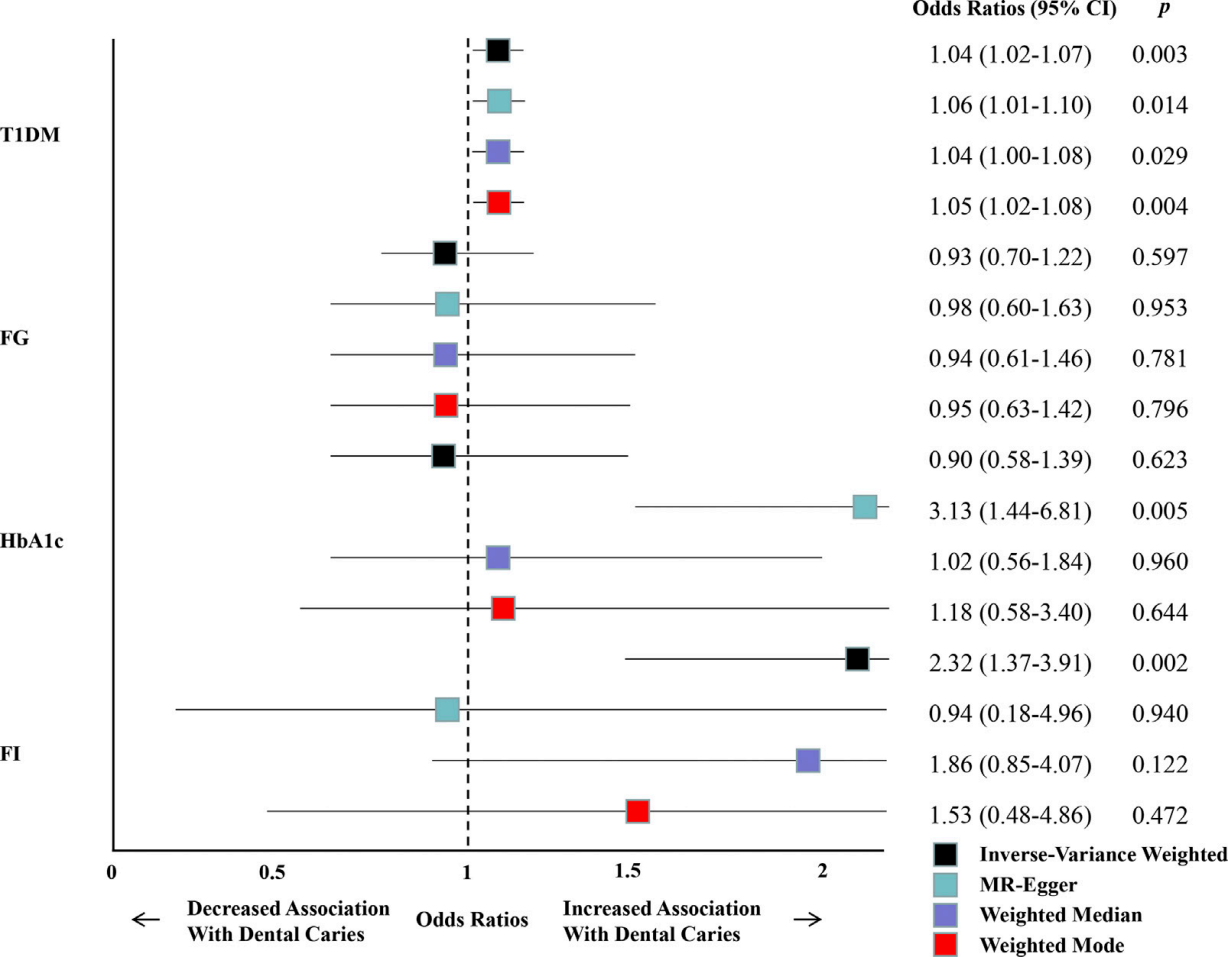
Summary of univariable MR analyses of the relationship between T1DM, glycemic traits, and the risk of dental caries. MR analyses (IVW, MR-Egger, weighted mode, and median weighted) of the association between genetically instrumented liability to T1DM, glycemic traits, and dental caries using variants from the GWAS summary statistics data.

**FIGURE 3:**
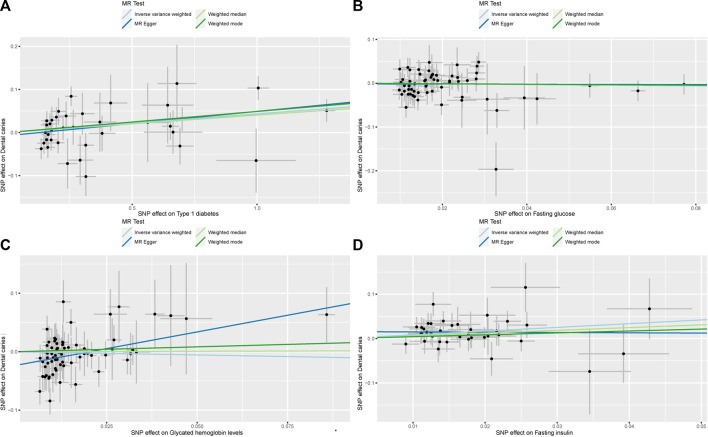
Scatterplot of the effect size for each SNP on T1DM, glycemic traits, and the risk of dental caries. **(A)** Scatterplot of the effect size for each SNP on T1DM and the risk of dental caries. **(B)** Scatterplot of the effect size for each SNP on FG and the risk of dental caries. **(C)** Scatterplot of the effect size for each SNP on HbA1c and the risk of dental caries. **(D)** Scatterplot of the effect size for each SNP on FI and the risk of dental caries.

A total of 39 SNPs of T1DM were chosen as instrumental variables after reviewing the GWAS summary statistics (Supplementary Table S3). Following the Mendelian randomization analysis with the TwoSampleMR package, univariable MR outcomes (IVW) revealed a significant causal relationship between T1DM and the increased risk of dental caries [odds ratio (OR) = 1.044; 95% confidence interval (CI) = 1.015–1.074; *p* = 0.003] (Figure 2) (Supplementary Table S8). Additionally, practically all supplementary methods revealed consistent connections for the effect of T1DM on dental caries risk (Figures 2, 3A) (Supplementary Table S8). In the Benjamini–Hochberg correction and Bonferroni correction of multiple testing, the *p*-values of the univariable MR outcomes (IVW) and other supplementary methods are still significant, except for the insignificant corrected *p*-value in the MR-Egger method by the Bonferroni correction (Table 1).

**FIGURE 4:**
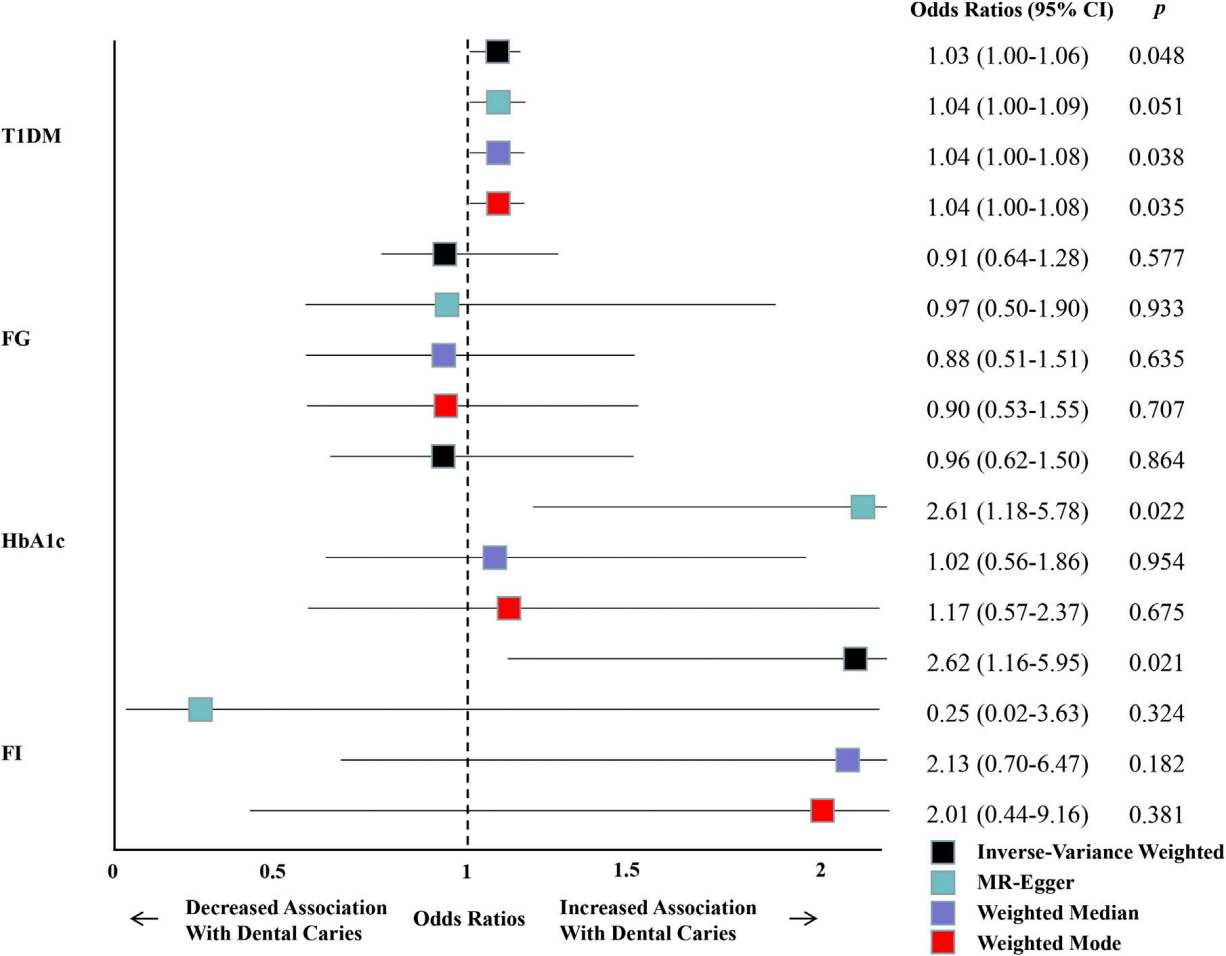
Summary of univariable MR analyses of the relationship between T1DM, glycemic traits, and the risk of dental caries after excluding SNPs that may be considered confounders according to our findings.

According to the PhenoScanner search (Supplementary Table S9) and leave-one-out results (Supplementary Figure S1), we selected and then excluded one outlier site SNP (rs506770) and five SNPs (rs9296062, rs6909461, rs6679677, rs1131017, and rs10774624), which may be considered confounders according to our findings (Supplementary Table S7). We performed a sensitivity analysis by excluding these six SNPs in T1DM and found an estimate similar to the original IVW analysis (OR = 1.030; 95% CI = 1.000–1.060; *p* = 0.048) and all the supplementary methods (Figure 4) (Supplementary Table S10). Additionally, the forest plots and funnel plots demonstrated that there was no discernible variability among the chosen SNPs for the instrumental variable (Supplementary Figures S2, S3).

A heterogeneity analysis was then conducted, and the results revealed significant heterogeneity in the effect of T1DM on dental caries (MR-Egger *p* = 0.040, IVW *p* = 0.042) (Supplementary Table S11). The Egger intercept showed that there was no horizontal pleiotropy in the effect of T1DM on dental caries (Egger intercept *p* = 0.428), and the MR-PRESSO analysis showed that horizontal pleiotropy was significant in the effect of T1DM on dental caries (global test *p* = 0.032) (Supplementary Table S12). However, when we excluded the six SNPs (rs506770, rs9296062, rs6909461, rs6679677, rs1131017, and rs10774624) in T1DM, as described in the previous sensitivity analysis, the heterogeneity and horizontal pleiotropy both disappeared (MR-Egger *p* = 0.434, IVW *p* = 0.440) (Egger intercept *p* = 0.356, global test *p* = 0.350) (Supplementary Tables S13, S14).

**FIGURE 5:**
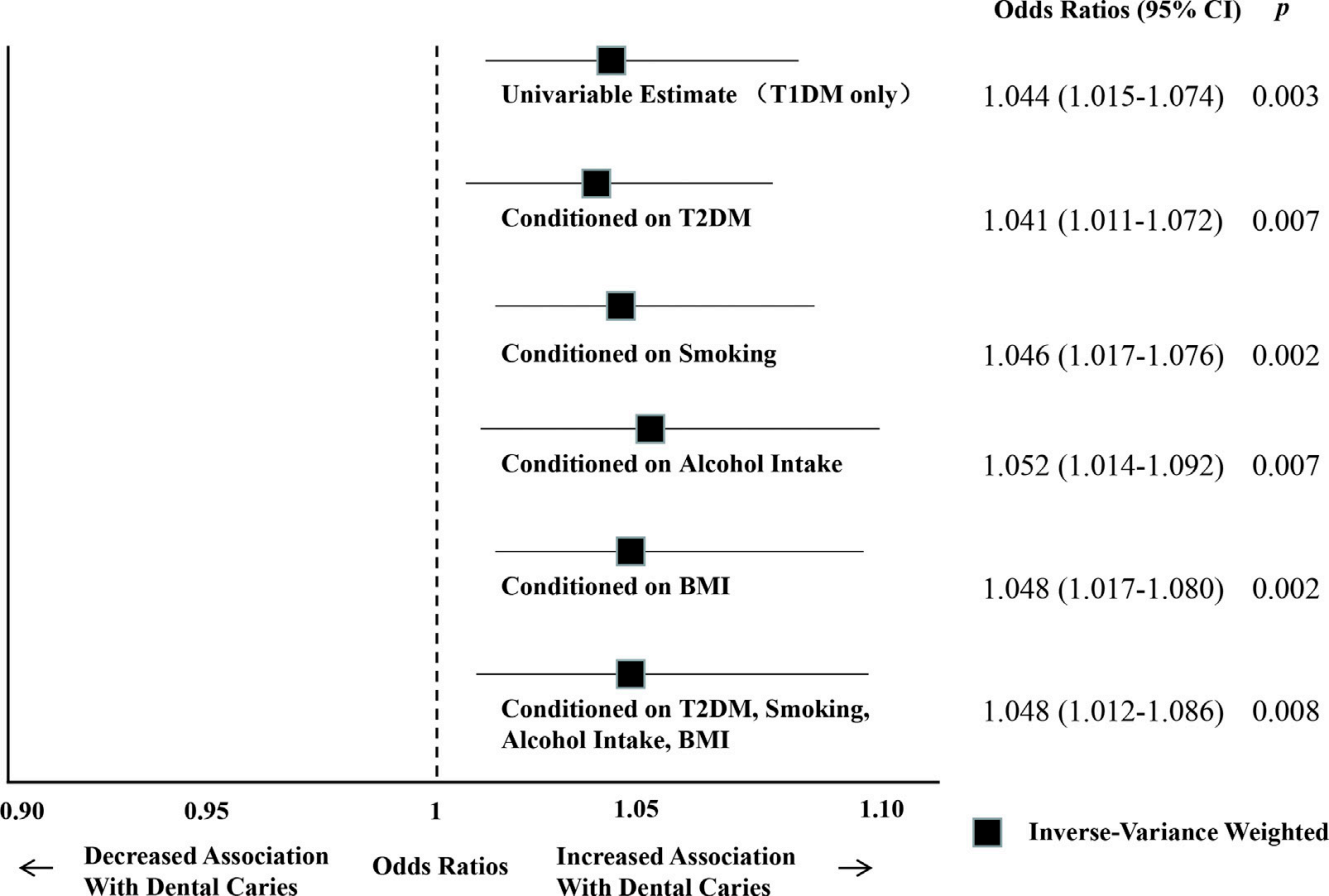
Summary of multivariable MR analyses of the relationship between T1DM and dental caries. Multivariable MR analyses (IVW) included potential mediators or confounders, such as T2DM, alcohol intake, and BMI, or all of these traits.

Furthermore, in the multivariable MR analysis (IVW), estimates similar to the original IVW analysis (OR = 1.044; 95% CI = 1.015–1.074; *p* = 0.003) were obtained when potential mediators or confounders, such as T2DM (OR = 1.041; 95% CI = 1.011–1.072; *p* = 0.007), smoking (OR = 1.046; 95% CI = 1.017–1.076; *p* = 0.002), alcohol intake (OR = 1.052; 95% CI = 1.014–1.092; *p* = 0.007), and BMI (OR = 1.048; 95% CI = 1.017–1.080; *p* = 0.002), or all of the abovementioned traits (OR = 1.048; 95% CI = 1.012–1.086; *p* = 0.008), were included in the model (Figure 5; Supplementary Table S15).

### Genetic association between glycemic traits and dental caries

The SNPs of glycemic traits that met the selection criteria (66 SNPs for FG, 73 SNPs for HbA1c, and 38 SNPs for FI) were chosen as instrumental variables after reviewing the GWAS summary statistics (Supplementary Tables S4–S6). Following the Mendelian randomization analysis with the TwoSampleMR package, IVW outcomes revealed a non-significant causal relationship between FG and HbA1c and an increased risk of dental caries (FG: OR = 0.928; 95% CI = 0.703–1.225; *p* = 0.597 and HbA1c: OR = 0.896; 95% CI = 0.578–1.388; *p* = 0.623) (Figure 2, 3B, C) (Supplementary Table S8). Additionally, although the IVW outcomes revealed a significant causal relationship between FI and an increased risk of dental caries (OR = 2.318; 95% CI = 1.375–3.908; *p* = 0.002), the other supplementary methods showed inconsistent connections for the effect of FI on the risk of dental caries (Figure 2, 3D) (Supplementary Table S8).

According to the PhenoScanner search (Supplementary Tables S16–S18) and leave-one-out results (Supplementary Figures S4–S6), we also selected and then excluded outlier SNPs of glycemic traits (Supplementary Table S7), which may be considered confounders according to our findings. Then, we conducted the second Mendelian randomization analysis after excluding these outlier SNPs of glycemic traits. When compared with the initial analysis, the IVW outcomes still revealed a non-significant causal relationship between FG and HbA1c and an increased risk of dental caries (FG: OR = 0.906; 95% CI = 0.639–1.283; *p* = 0.577 and HbA1c: OR = 0.962; 95% CI = 0.617–1.499; *p* = 0.864) (Figure 4) (Supplementary Table S10). Additionally, the outcomes of FI were also consistent with the results of the first MR analysis. The IVW outcomes were significant (OR = 2.621; 95% CI = 1.156–5.946; *p* = 0.021), while the other supplementary methods were not significant (Figure 4) (Supplementary Table S10).

A heterogeneity analysis was then conducted, and the results revealed no significant heterogeneity between the effect of glycemic traits on dental caries (FG: MR-Egger *p* = 0.447, IVW *p* = 0.480; HbA1c: MR-Egger *p* = 0.436, IVW *p* = 0.119; and FI: MR-Egger *p* = 0.879, IVW *p* = 0.867) (Supplementary Table S10). The Egger intercept showed that there was no horizontal pleiotropy between the effect of FG and FI on dental caries (Egger intercept *p* = 0.781 and Egger intercept *p* = 0.269), while there was horizontal pleiotropy between the effect of HbA1c on dental caries (Egger intercept *p* = 0.00045), and the MR-PRESSO analysis showed that horizontal pleiotropy was not significant between the effects of FG, HbA1c, and FI on dental caries (global test *p* = 0.593, global test *p* = 0.482, global test *p* = 0.893, and global test *p* = 0.870) (Supplementary Table S12).

### Genetic association between T1DM, glycemic traits, and progression of dental caries in replication cohort

Supplementary Tables S19–S22 summarize all the instrumental variables chosen for the replication cohort after reviewing the GWAS summary statistics. Univariable MR outcomes (IVW) revealed a significant causal relationship between T1DM and the increased rate of progression of dental caries (*β* = 0.015, 95% CI: 0.011 to 0.019, and *p* < 0.001) (Supplementary Table S23), with consistent findings in three other MR methods. In addition, the main IVW analyses did not support a causal effect of glycemic traits on the progression of dental caries (FG: *β* = 0.027, 95% CI: −0.038 to 0.091, *p* = 0.423; HbA1c: *β* = 0.050, 95% CI: −0.031 to 0.131, *p* = 0.225; and FI: *β* = −0.015, 95% CI: −0.108 to 0.078, *p* = 0.755).

## Discussion

In this study, we applied four MR methods to evaluate the effects of both T1DM and glycemic traits on the risk of dental caries, and we consider IVW to have higher statistical power than other MR approaches (Lin et al., 2021). Furthermore, other MR methods, such as MR-Egger, weighted mode, and median weighted mode, were implemented to ensure the robustness of IVW estimates, as previous studies have shown (Zhao et al., 2022).

First, we determined that T1DM could increase the risk of dental caries, as IVW outcomes revealed a significant causal relationship between T1DM and the increased risk of dental caries (OR = 1.044; 95% CI = 1.015–1.074; and *p* = 0.003) (Figure 2) (Supplementary Table S8). Additionally, practically all supplementary methods and the replication cohort revealed consistent connections for the effect of T1DM on the development and progression of dental caries (Figures 2, 3A) (Supplementary Tables S8, S23), and the corrected *p*-values of almost all multiple testing results still showed statistical differences (Table 1). However, because the IVW outcomes revealed a non-significant causal relationship between FG and HbA1c and the increased risk of dental caries (FG: OR = 0.928; 95% CI = 0.703–1.225; *p* = 0.597 and HbA1c: OR = 0.896; 95% CI = 0.578–1.388; *p* = 0.623), only the IVW outcomes revealed a significant causal relationship between FI and the increased risk of dental caries (OR = 2.318; 95% CI = 1.375–3.908; *p* = 0.002), with non-significant results being observed in the supplementary methods (Figure 2) (Supplementary Table S8). In addition, the main IVW analyses of the replication cohort also did not support a causal effect of glycemic traits on the progression of dental caries (Supplementary Table S23). We conclude that the causal relationship between glycemic traits and dental caries does not exist according to our results.

Then, we conducted a sensitivity analysis to identify the potential confounding factors, heterogeneity, and horizontal pleiotropy in our study on the causal relationship between T1DM and dental caries. The initial MR analysis revealed heterogeneity and pleiotropy (*p* < 0.05) (Supplementary Tables S11, S12). However, after excluding one outlier site SNP (rs506770) and five additional SNPs (rs9296062, rs6909461, rs6679677, rs1131017, and rs10774624), which we identified as confounding variables based on our findings (Supplementary Table S7), the heterogeneity and horizontal pleiotropy both disappeared (*p* > 0.05) (Supplementary Tables S13, S14). In addition, in the second analysis that excluded the confounding SNPs (Figure 4) (Supplementary Table S10), or the multivariable MR analysis that included potential mediators or confounders, such as T2DM, alcohol intake, and BMI, or all of these traits (Figure 5) (Supplementary Table S15), both the estimates were similar to the original IVW analysis, further confirming the stability of our results. In terms of the MR analysis of the causal relationship between glycemic traits and dental caries, the results revealed no significant heterogeneity and horizontal pleiotropy between all the glycemic traits and dental caries (*p* > 0.05), except for horizontal pleiotropy that was found between HbA1c and dental caries. At present, it is generally believed that when horizontal pleiotropy cannot be avoided, the results of MR-Egger are more convincing than those of IVW because the intercept is considered in the regression model of the MR-Egger calculation function, the main purpose being to judge whether there is horizontal pleiotropy (Burgess and Thompson, 2017). Therefore, we should have taken IVW as the main MR method for glycemic traits of FG and FI and MR-Egger for glycemic traits of HbA1c. However, based on the inconsistency of the four MR methods in glycemic traits before and after excluding confounding SNPs (Figures 2, 4) (Supplementary Tables S8, S10), we concluded that there is no obvious causal relationship between all the glycemic traits and dental caries because all the MR methods may explain the reliability of the conclusion to a certain extent (Zhao et al., 2022). In summary, our study determined that T1DM was related to a high risk of dental caries, while glycemic traits (FG, HbA1c, and FI) were not associated with the risk of dental caries.

Our MR study has shown that there is a relationship between genetic variants predisposing patients to T1DM and dental caries. However, we found no evidence of a causal relationship between FG, HbA1c, or FI and the risk of dental caries. This may reflect that the pathophysiological pathways between T1DM and dental caries are independent of glycemic traits at the genetic level. First, we propose this hypothesis based on the fact that glycemic traits are not a unique feature of diabetes. For example, people with several other illnesses, such as pancreatitis, stroke, and cardiovascular diseases, can exhibit significant changes in glycemic traits, which may be mediated by a complicated interaction of inflammatory pathways, glucoregulatory hormones, and neuroendocrine systems, even if diabetes does not exist in these individuals (Shi et al., 2021; Bharmal et al., 2022; Tao et al., 2022). Additionally, in traditional observational studies, when studying the relationship between T1DM and dental caries, glycemic traits, such as HbA1c, are usually combined as indicators to measure the severity of T1DM (Akpata et al., 2012; Pachoński et al., 2020). However, changes in glycemic traits are not the only feature of T1DM. For instance, current research shows that T1DM patients have low levels of 25-hydroxyvitamin D (25OHD), a routinely tested vitamin D metabolite in blood, regardless of the disease’s glycemic traits (Raab et al., 2014; Liu et al., 2018). Additionally, a study on newborns has suggested that 25OHD gene expression dysregulation may serve as latent cues for the advancement of T1DM (Li et al., 2021). Furthermore, numerous investigations have demonstrated an inverse relationship between serum 25OHD and dental caries because serum 25OHD can enhance the absorption of calcium and phosphorus and promote the mineralization of the hydroxyapatite crystal structure in teeth (Schroth et al., 2016; Kim et al., 2018). In light of these viewpoints, it is possible to explain the finding that the pathophysiological link between T1DM and dental caries is unrelated to glycemic traits.

In addition, previous studies have speculated that glycemic traits are correlated with dental caries because it is thought that the increase in blood glucose in T1DM would also bring about a rise in saliva glucose levels, which would alter the efficacy of salivary protection against dental caries (Miko et al., 2010; Sampaio et al., 2011; Ahmad and Haque, 2021). This is based on physiological assumptions that increased glucose content in the entire saliva is a direct reflection of the blood glucose levels that are derived from the ultrafiltrate of the plasma through three mechanisms: passive diffusion, active transport, and ultrafiltration (Miller, 1993). However, the aforementioned theory is still debatable because other research has shown that saliva and blood glucose levels in T1DM patients are unrelated, as saliva has the capacity to eliminate exogenous glucose (Goulet et al., 1985; Borg Andersson et al., 1998; Jurysta et al., 2009; Lima-Aragão et al., 2016). Therefore, although the results of our MR analysis support that there is no significant relationship between glycemic traits and dental caries, contradictions in the observation study imply that the relationship between glycemic traits and dental caries is still uncertain and requires further discussion in the future.

Furthermore, the primary reason for T1DM promoting the risk of dental caries is likely its adverse effect on saliva function. Many studies have indicated that individuals with T1DM experience reduced salivary gland function, resulting in significantly less saliva secretion during rest and stimulation (Ferizi et al., 2018; Pappa et al., 2021). Due to this reduction in the function of the salivary gland in T1DM patients during the development of dental caries, there is a decrease in not only the protective mechanical rinsing effect that could play a role in preventing dental caries (Hicks et al., 2003) but also in the levels of various saliva-secreting proteins that play antimicrobial, anti-demineralization, and immune monitoring roles (Cabras et al., 2010; Gao et al., 2016; Hegde et al., 2019). This significantly elevates the risk of dental caries in individuals with T1DM. Therefore, based on the results of this study and the close associations between diabetes, bacteria, and dental caries (Arora et al., 2021; Carelli et al., 2023), the necessity of preventing dental caries is highlighted, especially in patients with T1DM. Measures such as adding antibacterial and anti-diabetic substances to the diet or cultivating good oral hygiene habits may be of significant importance, particularly in children (Soares et al., 2021; Tit and Bungau, 2023).

Overall, our MR study shows that there is a strong link between genetic variants predisposing patients to T1DM and a high risk of dental caries. This suggests that the periodic reinforcement of oral hygiene instructions has to be added to the management and early multidisciplinary intervention of T1DM patients, especially among adolescents and teenagers, who are more susceptible to T1DM. Moreover, our research still has many limitations. The first limitation of this study is that covariate adjustment is often used in MR studies to reduce the influence of confounding variables and improve the accuracy of the results. However, if the adjusted covariates include confounding variables related to dental caries, such as food intake and oral hygiene habits, then collider bias may be introduced during the covariate adjustment. For example, the GWAS data that we used for glycemic traits adjusted for BMI, and if there is a relationship between the confounding variables and both BMI and dental caries, then the adjustment may mask the influence of these confounding variables on the causal relationship between glycemic traits and dental caries, thereby affecting the stability of the conclusion. Therefore, further clinical cohort studies may be necessary in the future to confirm the reliability of the negative results between glycemic traits and dental caries that we obtained. Second, our research is limited to European populations, and it may not be scalable to other races. Third, we may have overlooked some instrumental variables related to potential confounding factors, which may violate the basic assumptions of the MR analysis. Finally, the risk of dental caries is determined by both genetic and environmental factors, and our findings only partially address the genetic influence of T1DM on dental caries.

## Conclusion

In conclusion, this is the first MR study that evaluated the causal effect of T1DM on dental caries. Moreover, because T1DM contributes to dental caries at the genetic level, this suggests that the periodic reinforcement of oral hygiene instructions has to be added to the management and early multidisciplinary intervention of T1DM patients, especially among adolescents and teenagers, who are more susceptible to T1DM.

## Data Availability

The original contributions presented in the study are included in the article/Supplementary Material; further inquiries can be directed to the corresponding authors.
