# Supplementary material for: Type 1 diabetes, glycemic traits, and risk of dental caries: a Mendelian randomization study
Source: Front Genet. 2023 Oct 10;14:1230113. doi: 10.3389/fgene.2023.1230113 (PMC10597668; doi:10.3389/fgene.2023.1230113)
Supplement: Supplementary file 1 [file DataSheet1.ZIP › Supplementary Table S10.docx]

**Table S10** The second MR analysis results of four common methods of T1DM and glycemic traits to dental caries.

| Exposure Source | Outcome Source | Outcome | Exposure | Method | nsnp | b | se | p | OR |
| --- | --- | --- | --- | --- | --- | --- | --- | --- | --- |
| PMID: 32005708 | The FinnGen Biobank | Dental caries | T1DM | IVW | 33 | 0.029 | 0.015 | 0.048 | 1.030 |
| PMID: 32005708 | The FinnGen Biobank | Dental caries | T1DM | MR Egger | 33 | 0.044 | 0.022 | 0.051 | 1.045 |
| PMID: 32005708 | The FinnGen Biobank | Dental caries | T1DM | Weighted median | 33 | 0.040 | 0.019 | 0.038 | 1.041 |
| PMID: 32005708 | The FinnGen Biobank | Dental caries | T1DM | Weighted mode | 33 | 0.039 | 0.018 | 0.035 | 1.040 |
| PMID: 34059833 | The FinnGen Biobank | Dental caries | FG | IVW | 54 | -0.099 | 0.178 | 0.577 | 0.906 |
| PMID: 34059833 | The FinnGen Biobank | Dental caries | FG | MR Egger | 54 | -0.029 | 0.343 | 0.933 | 0.971 |

**Table 10** Continued.

| Exposure Source | Outcome Source | Outcome | Exposure | Method | nsnp | b | se | p | OR |
| --- | --- | --- | --- | --- | --- | --- | --- | --- | --- |
| PMID: 34059833 | The FinnGen Biobank | Dental caries | FG | Weighted median | 54 | -0.131 | 0.277 | 0.635 | 0.877 |
| PMID: 34059833 | The FinnGen Biobank | Dental caries | FG | Weighted mode | 54 | -0.104 | 0.275 | 0.707 | 0.901 |
| PMID: 34059833 | The FinnGen Biobank | Dental caries | HbA1c | IVW | 60 | -0.039 | 0.226 | 0.864 | 0.962 |
| PMID: 34059833 | The FinnGen Biobank | Dental caries | HbA1c | MR Egger | 60 | 0.958 | 0.406 | 0.022 | 2.607 |
| PMID: 34059833 | The FinnGen Biobank | Dental caries | HbA1c | Weighted median | 60 | 0.018 | 0.307 | 0.954 | 1.018 |
| PMID: 34059833 | The FinnGen Biobank | Dental caries | HbA1c | Weighted mode | 60 | 0.153 | 0.363 | 0.675 | 1.165 |

**Table 10** Continued.

| Exposure Source | Outcome Source | Outcome | Exposure | Method | nsnp | b | se | p | OR |
| --- | --- | --- | --- | --- | --- | --- | --- | --- | --- |
| PMID: 34059833 | The FinnGen Biobank | Dental caries | FI | IVW | 18 | 0.964 | 0.418 | 0.021 | 2.621 |
| PMID: 34059833 | The FinnGen Biobank | Dental caries | FI | MR Egger | 18 | -1.392 | 1.368 | 0.324 | 0.249 |
| PMID: 34059833 | The FinnGen Biobank | Dental caries | FI | Weighted median | 18 | 0.756 | 0.567 | 0.182 | 2.130 |
| PMID: 34059833 | The FinnGen Biobank | Dental caries | FI | Weighted mode | 18 | 0.696 | 0.775 | 0.381 | 2.006 |
