# Supplementary material for: Type 1 diabetes, glycemic traits, and risk of dental caries: a Mendelian randomization study
Source: Front Genet. 2023 Oct 10;14:1230113. doi: 10.3389/fgene.2023.1230113 (PMC10597668; doi:10.3389/fgene.2023.1230113)
Supplement: Supplementary file 1 [file DataSheet1.ZIP › Supplementary Table S2 .docx]

**Table S2** Basic information on the GWAS applied in this study.

| GWAS Source | Year | Trait | Sample Size | Population | | Number of SNPs |
| --- | --- | --- | --- | --- | --- | --- |
| PMID: 32005708 | 2020 | T1DM | 24,840 | European | | 12,783,129 |
| PMID: 34059833 | 2021 | FG | 336,639 | | European | 10,894,596 |
| PMID: 34059833 | 2021 | HbA1c | 146,806 | | European | 30,649,064 |
| PMID: 34059833 | 2021 | FI | 151,013 | | European | 29,664,438 |
| The FinnGen Biobank | 2021 | Dental caries | 199,565 | | European | 16,380,411 |
