# Supplementary material for: Type 1 diabetes, glycemic traits, and risk of dental caries: a Mendelian randomization study
Source: Front Genet. 2023 Oct 10;14:1230113. doi: 10.3389/fgene.2023.1230113 (PMC10597668; doi:10.3389/fgene.2023.1230113)
Supplement: Supplementary file 1 [file DataSheet1.ZIP › Supplementary Table S7.docx]

**Table S7** Summary of association (P < 5x10^-8^) of the SNPs used as instruments (Type 1 diabetes mellitus and glycemic traits ) in the primary analysis with confounders or outcome risk factors in PhenoScanner (Search parameter settings : Catalogue = Diseases&traits, p-value = 5x10^-8^ Proxie s= none, r^2^=0.8, Build = 37) (accessed on 2023/02/11) and outlier SNP of leave−one−out sensitivity analysis.

| SNP | Trait | BETA | SE | P | PMID | Exposure |
| --- | --- | --- | --- | --- | --- | --- |
| rs9296062 | Rheumatoid arthritis | 0.3011 | 0.03453 | 4.00E-18 | 24390342 | T1DM |
| rs6909461 | Trunk fat-free mass | 0.01214 | 0.001843 | 4.49E-11 | UKBB | T1DM |
| rs6909461 | Trunk predicted mass | 0.01209 | 0.001837 | 4.60E-11 | UKBB | T1DM |
| rs6909461 | Whole body fat-free mass | 0.0107 | 0.00185 | 7.41E-09 | UKBB | T1DM |
| rs6909461 | Whole body water mass | 0.01066 | 0.001853 | 8.90E-09 | UKBB | T1DM |
| rs6909461 | Rheumatoid arthritis | -0.1625 | 0.02688 | 1.60E-08 | 24390342 | T1DM |
| rs6679677 | Rheumatoid arthritis | 0.6831 | 0.06622 | 6.00E-25 | 17554300 | T1DM |
| rs1131017 | Leg fat-free mass left | -0.009046 | 0.001616 | 2.16E-08 | UKBB | T1DM |
| rs1131017 | Leg fat-free mass right | -0.008993 | 0.001615 | 2.57E-08 | UKBB | T1DM |
| rs1131017 | Leg predicted mass left | -0.008925 | 0.001605 | 2.67E-08 | UKBB | T1DM |
| rs1131017 | Leg predicted mass right | -0.008873 | 0.001605 | 3.20E-08 | UKBB | T1DM |
| rs1131017 | Trunk fat-free mass | -0.009875 | 0.001545 | 1.66E-10 | UKBB | T1DM |
| rs1131017 | Trunk predicted mass | -0.009787 | 0.00154 | 2.11E-10 | UKBB | T1DM |
| rs1131017 | Whole body fat-free mass | -0.009732 | 0.001552 | 3.56E-10 | UKBB | T1DM |
| rs1131017 | Whole body water mass | -0.009617 | 0.001554 | 6.06E-10 | UKBB | T1DM |
| rs1131017 | Rheumatoid arthritis | -0.07696 | 0.01418 | 1.30E-08 | 24390342 | T1DM |
| rs1131017 | Years of educational attainment | 0.014 | 0.003 | 1.81E-08 | 27225129 | T1DM |
| rs10774624 | Past tobacco smoking | 0.01919 | 0.003199 | 2.00E-09 | UKBB | T1DM |
| rs10774624 | Smoking status: previous | -0.00687 | 0.001175 | 5.00E-09 | UKBB | T1DM |
| rs10774624 | Weight | 0.01336 | 0.002144 | 4.70E-10 | UKBB | T1DM |
| rs10774624 | Whole body fat-free mass | 0.014 | 0.001551 | 1.81E-19 | UKBB | T1DM |
| rs10774624 | Whole body water mass | 0.01411 | 0.001554 | 1.05E-19 | UKBB | T1DM |
| rs10774624 | Hip circumference | 0.01573 | 0.00243 | 9.49E-11 | UKBB | T1DM |
| rs10774624 | Rheumatoid arthritis | NA | NA | 7.00E-09 | 24390342 | T1DM |
| rs506770 | Outlier SNP of MR leave−one−out sensitivity analysis | |  |  |  | T1DM |

**Table S7** Continued.

| SNP | Trait | BETA | SE | P | PMID | Exposure |
| --- | --- | --- | --- | --- | --- | --- |
| rs10811660 | Body mass index | 0.03557 | 0.00396 | 2.69E-19 | 28892062 | FG |
| rs10830963 | Obesity related traits | NA | NA | 4.00E-08 | 23251661 | FG |
| rs11708067 | Body mass index | -0.016 | 0.0027 | 8.90E-09 | 29273807 | FG |
| rs11708067 | Body mass index | -0.014 | 0.0025 | 2.00E-08 | 29273807 | FG |
| rs1260326 | Alcohol consumption | -0.028 | 0.002924 | 1.00E-21 | 28937693 | FG |
| rs1260326 | Alcohol consumption in current drinkers | 0.03 | 0.002977 | 7.00E-24 | 28937693 | FG |
| rs1260326 | Alcohol intake frequency | -0.048 | 0.003633 | 7.60E-40 | UKBB | FG |
| rs1260326 | Alcohol intake versus 10 years previously | -0.01242 | 0.001887 | 4.75E-11 | UKBB | FG |
| rs1260326 | Arm fat-free mass left | 0.01965 | 0.001579 | 1.49E-35 | UKBB | FG |
| rs1260326 | Arm fat-free mass right | 0.02004 | 0.001547 | 2.28E-38 | UKBB | FG |
| rs1260326 | Arm predicted mass left | 0.01948 | 0.001573 | 3.23E-35 | UKBB | FG |
| rs1260326 | Arm predicted mass right | 0.01989 | 0.001542 | 4.46E-38 | UKBB | FG |
| rs1260326 | Weight | 0.01778 | 0.00217 | 2.53E-16 | UKBB | FG |
| rs1260326 | Whole body fat-free mass | 0.02108 | 0.00157 | 4.40E-41 | UKBB | FG |
| rs1260326 | Whole body water mass | 0.02132 | 0.001572 | 7.14E-42 | UKBB | FG |
| rs1604038 | Body mass index | -0.01869 | 0.002661 | 2.17E-12 | UKBB | FG |
| rs1604038 | Hip circumference | -0.01713 | 0.002664 | 1.27E-10 | UKBB | FG |
| rs1604038 | Leg fat mass left | -0.01304 | 0.002126 | 8.76E-10 | UKBB | FG |
| rs1604038 | Leg fat mass right | -0.01278 | 0.002151 | 2.81E-09 | UKBB | FG |
| rs1604038 | Trunk fat mass | -0.01583 | 0.002709 | 5.05E-09 | UKBB | FG |
| rs1604038 | Waist circumference | -0.01493 | 0.002382 | 3.67E-10 | UKBB | FG |
| rs1604038 | Weight | -0.01538 | 0.00235 | 6.09E-11 | UKBB | FG |
| rs1604038 | Whole body fat mass | -0.01569 | 0.002625 | 2.26E-09 | UKBB | FG |
| rs16851397 | Whole body fat-free mass | -0.02021 | 0.003586 | 1.75E-08 | UKBB | FG |
| rs16851397 | Whole body water mass | -0.02021 | 0.003591 | 1.83E-08 | UKBB | FG |
| rs17265513 | Whole body fat mass | 0.01668 | 0.002962 | 1.78E-08 | UKBB | FG |

**Table S7** Continued.

| SNP | Trait | BETA | SE | P | PMID | Exposure |
| --- | --- | --- | --- | --- | --- | --- |
| rs1820176 | Hip circumference | -0.01582 | 0.002669 | 3.08E-09 | UKBB | FG |
| rs1820176 | Whole body fat mass | -0.0146 | 0.002631 | 2.85E-08 | UKBB | FG |
| rs1820176 | Whole body fat-free mass | -0.01501 | 0.001705 | 1.32E-18 | UKBB | FG |
| rs1820176 | Whole body water mass | -0.01503 | 0.001707 | 1.30E-18 | UKBB | FG |
| rs2238435 | Body mass index | -0.02536 | 0.002477 | 1.36E-24 | UKBB | FG |
| rs2238435 | Hip circumference | -0.02691 | 0.00248 | 1.99E-27 | UKBB | FG |
| rs2238435 | Waist circumference | -0.02079 | 0.002218 | 6.95E-21 | UKBB | FG |
| rs2238435 | Weight | -0.02607 | 0.002188 | 1.02E-32 | UKBB | FG |
| rs2238435 | Whole body fat mass | -0.02614 | 0.002443 | 1.05E-26 | UKBB | FG |
| rs2238435 | Whole body fat-free mass | -0.01735 | 0.001583 | 6.25E-28 | UKBB | FG |
| rs2238435 | Whole body water mass | -0.01726 | 0.001586 | 1.43E-27 | UKBB | FG |
| rs7012637 | Body mass index | 0.01624 | 0.002424 | 2.09E-11 | UKBB | FG |
| rs7903146 | Body mass index | 0.0234 | 0.0034 | 1.11E-11 | 25673413 | FG |
| rs7903146 | Waist circumference | 0.022 | 0.0036 | 1.70E-09 | 25673412 | FG |
| rs7903146 | Body mass index | -0.056 | 0.008209 | 9.00E-12 | 28892062 | FG |
| rs7903146 | Body mass index | 0.01569 | 0.002643 | 2.93E-09 | UKBB | FG |
| rs7903146 | Body mass index | -0.01494 | 0.0005723 | 3.72E-150 | UKBB | FG |
| rs7903146 | Body mass index | -0.005739 | 0.0006431 | 4.56E-19 | UKBB | FG |
| rs7903146 | Hip circumference | 0.02146 | 0.002646 | 5.10E-16 | UKBB | FG |
| rs7903146 | Whole body fat mass | 0.01486 | 0.002607 | 1.21E-08 | UKBB | FG |
| rs9348441 | Body mass index females | -0.03788 | 0.005442 | 3.36E-12 | 28892062 | FG |
| rs9348441 | Body mass index males | -0.04406 | 0.004978 | 8.64E-19 | 28892062 | FG |
| rs9348441 | Body mass index | -0.04076 | 0.003673 | 1.27E-28 | 28892062 | FG |
| rs10774624 | Rheumatoid arthritis | NA | NA | 7.00E-09 | 24390342 | HbA1c |
| rs10774624 | Hip circumference | 0.01573 | 0.00243 | 9.49E-11 | UKBB | HbA1c |
| rs10774624 | Leg fat-free mass left | 0.01164 | 0.001616 | 5.82E-13 | UKBB | HbA1c |

**Table S7** Continued.

| SNP | Trait | BETA | SE | P | PMID | Exposure |
| --- | --- | --- | --- | --- | --- | --- |
| rs10774624 | Leg fat-free mass right | 0.01221 | 0.001615 | 4.03E-14 | UKBB | HbA1c |
| rs10774624 | Leg predicted mass left | 0.01145 | 0.001605 | 9.75E-13 | UKBB | HbA1c |
| rs10774624 | Leg predicted mass right | 0.0121 | 0.001604 | 4.60E-14 | UKBB | HbA1c |
| rs10774624 | Past tobacco smoking | 0.01919 | 0.003199 | 2.00E-09 | UKBB | HbA1c |
| rs10774624 | Smoking status: previous | -0.00687 | 0.001175 | 5.00E-09 | UKBB | HbA1c |
| rs10774624 | Weight | 0.01336 | 0.002144 | 4.70E-10 | UKBB | HbA1c |
| rs10774624 | Whole body fat-free mass | 0.014 | 0.001551 | 1.81E-19 | UKBB | HbA1c |
| rs10774624 | Whole body water mass | 0.01411 | 0.001554 | 1.05E-19 | UKBB | HbA1c |
| rs10811661 | Body mass index | 0.03554 | 0.003961 | 2.93E-19 | 28892062 | HbA1c |
| rs10811661 | Body mass index | 0.021 | 0.002906 | 5.00E-13 | 28892062 | HbA1c |
| rs10946402 | Hip circumference | -0.01792 | 0.003186 | 1.85E-08 | UKBB | HbA1c |
| rs11257655 | Body mass index | 0.02142 | 0.003555 | 1.69E-09 | 28892062 | HbA1c |
| rs11257655 | Body mass index | -0.015 | 0.00259 | 7.00E-09 | 28892062 | HbA1c |
| rs117233107 | Weight | 0.07511 | 0.009165 | 2.50E-16 | UKBB | HbA1c |
| rs117233107 | Whole body fat-free mass | 0.07853 | 0.00663 | 2.34E-32 | UKBB | HbA1c |
| rs117233107 | Whole body water mass | 0.07781 | 0.00664 | 1.03E-31 | UKBB | HbA1c |
| rs12612492 | Whole body fat-free mass | 0.0166 | 0.002281 | 3.38E-13 | UKBB | HbA1c |
| rs12612492 | Whole body water mass | 0.0168 | 0.002285 | 1.91E-13 | UKBB | HbA1c |
| rs13234131 | Whole body fat-free mass | -0.01779 | 0.002289 | 7.83E-15 | UKBB | HbA1c |
| rs13234131 | Whole body water mass | -0.01798 | 0.002292 | 4.32E-15 | UKBB | HbA1c |
| rs1800562 | Alcohol consumption transferrin glycosylation | 0.629 | 0.05305 | 2.00E-32 | 21665994 | HbA1c |
| rs2001945 | Weight | 0.01541 | 0.002126 | 4.23E-13 | UKBB | HbA1c |
| rs2001945 | Whole body fat mass | 0.01567 | 0.002374 | 4.11E-11 | UKBB | HbA1c |
| rs204995 | Rheumatoid arthritis | NA | NA | 7.74E-48 | 20453842 | HbA1c |
| rs204995 | Weight | 0.02029 | 0.002409 | 3.81E-17 | UKBB | HbA1c |
| rs204995 | Whole body fat-free mass | 0.01738 | 0.001743 | 2.06E-23 | UKBB | HbA1c |

**Table S7** Continued.

| SNP | Trait | BETA | SE | P | PMID | Exposure |
| --- | --- | --- | --- | --- | --- | --- |
| rs204995 | Whole body water mass | 0.01731 | 0.001746 | 3.74E-23 | UKBB | HbA1c |
| rs204995 | Rheumatoid arthritis | -0.392 | 0.01903 | 7.10E-85 | 24390342 | HbA1c |
| rs204995 | Rheumatoid arthritis | -0.2151 | 0.01646 | 3.80E-39 | 24390342 | HbA1c |
| rs204995 | Rheumatoid arthritis | -0.4308 | 0.03144 | 7.74E-48 | 20453842 | HbA1c |
| rs6804915 | Waist circumference | 0.01426 | 0.002371 | 1.81E-09 | UKBB | HbA1c |
| rs6804915 | Weight | 0.01477 | 0.002339 | 2.73E-10 | UKBB | HbA1c |
| rs6804915 | Whole body fat mass | 0.01479 | 0.002612 | 1.50E-08 | UKBB | HbA1c |
| rs7903146 | Waist circumference | 0.022 | 0.0037 | 3.90E-09 | 25673412 | HbA1c |
| rs7903146 | Waist circumference | 0.022 | 0.0036 | 1.70E-09 | 25673412 | HbA1c |
| rs7903146 | Obesity in type 2 diabetes body mass index | NA | NA | 1.40E-08 | 19056611 | HbA1c |
| rs7903146 | Type 2 diabetes with obesity | NA | NA | 4.34E-10 | 20581827 | HbA1c |
| rs7903146 | Body mass index | 0.023 | 0.003379 | 1.00E-11 | 25673413 | HbA1c |
| rs7903146 | Body mass index | 0.024 | 0.003366 | 1.00E-12 | 25673413 | HbA1c |
| rs7903146 | Weight | 0.01288 | 0.002335 | 3.42E-08 | UKBB | HbA1c |
| rs7903146 | Whole body fat mass | 0.01486 | 0.002607 | 1.21E-08 | UKBB | HbA1c |
| rs9818758 | Years of educational attainment | 0.018 | 0.003 | 3.99E-08 | 27225129 | HbA1c |
| rs10050393 | Body fat percentage | 0.01053 | 0.001875 | 1.96E-08 | UKBB | FI |
| rs10865959 | Waist circumference | 0.01519 | 0.002304 | 4.32E-11 | UKBB | FI |
| rs10865959 | Weight | 0.015 | 0.002273 | 4.17E-11 | UKBB | FI |
| rs10865959 | Whole body fat mass | 0.01952 | 0.002538 | 1.49E-14 | UKBB | FI |
| rs116141873 | Waist circumference | -0.02991 | 0.005035 | 2.84E-09 | UKBB | FI |
| rs11708067 | Body mass index | -0.016 | 0.0027 | 8.90E-09 | 29273807 | FI |
| rs11708067 | Body mass index | -0.014 | 0.0025 | 2.00E-08 | 29273807 | FI |
| rs11727676 | Body mass index | -0.0358 | 0.0064 | 2.55E-08 | 25673413 | FI |
| rs11727676 | Body mass index | -0.0365 | 0.0063 | 6.25E-09 | 25673413 | FI |
| rs11727676 | Body mass index | -0.037 | 0.006361 | 6.00E-09 | 25673413 | FI |

**Table S7** Continued.

| SNP | Trait | BETA | SE | P | PMID | Exposure |
| --- | --- | --- | --- | --- | --- | --- |
| rs11727676 | Body mass index | -0.036 | 0.006497 | 3.00E-08 | 25673413 | FI |
| rs1206760 | Hip circumference | 0.0157 | 0.002433 | 1.11E-10 | UKBB | FI |
| rs12454712 | Body mass index | 0.018 | 0.002708 | 3.00E-11 | 28892062 | FI |
| rs12454712 | Waist to hip ratio adjusted for body mass index | -0.0347 | 0.00568 | 1.00E-09 | 25673412 | FI |
| rs12454712 | Waist to hip ratio adjusted for body mass index | -0.0329 | 0.005547 | 3.00E-09 | 25673412 | FI |
| rs12454712 | Body mass index | 0.01432 | 0.002479 | 7.62E-09 | UKBB | FI |
| rs12454712 | Hip circumference | 0.01857 | 0.002482 | 7.31E-14 | UKBB | FI |
| rs12454712 | Weight | 0.01386 | 0.00219 | 2.47E-10 | UKBB | FI |
| rs12454712 | Whole body fat-free mass | 0.009324 | 0.001585 | 4.03E-09 | UKBB | FI |
| rs12454712 | Whole body water mass | 0.009472 | 0.001587 | 2.41E-09 | UKBB | FI |
| rs1260326 | Alcohol consumption | -0.028 | 0.002924 | 1.00E-21 | 28937693 | FI |
| rs1260326 | Alcohol consumption in current drinkers | 0.03 | 0.002977 | 7.00E-24 | 28937693 | FI |
| rs1260326 | Alcohol intake frequency | -0.048 | 0.003633 | 7.60E-40 | UKBB | FI |
| rs1260326 | Alcohol intake versus 10 years previously | -0.01242 | 0.001887 | 4.75E-11 | UKBB | FI |
| rs13389219 | Body mass index | -0.014 | 0.0023 | 2.60E-10 | 29273807 | FI |
| rs13389219 | Hip circumference | -0.021 | 0.0036 | 3.10E-09 | 25673412 | FI |
| rs13389219 | Waist hip ratio adjusted for smoking | 0.0292 | 0.0038 | 1.11E-14 | 28443625 | FI |
| rs13389219 | Waist hip ratio adjusted for smoking | 0.0289 | 0.0036 | 1.14E-15 | 28443625 | FI |
| rs13389219 | Waist hip ratio | NA | NA | 4.99E-10 | 20935629 | FI |
| rs13389219 | Waist hip ratio | 0.02 | 0.0034 | 2.50E-09 | 25673412 | FI |
| rs13389219 | Waist hip ratio | 0.02 | 0.0033 | 1.50E-09 | 25673412 | FI |
| rs13389219 | Whole body fat mass | -0.01906 | 0.002424 | 3.83E-15 | UKBB | FI |
| rs1351394 | Hip circumference | -0.023 | 0.0035 | 6.40E-11 | 25673412 | FI |
| rs1351394 | Hip circumference | -0.021 | 0.0035 | 7.10E-10 | 25673412 | FI |
| rs1351394 | Hip circumference adjusted for BMI | -0.024 | 0.0034 | 9.60E-13 | 25673412 | FI |
| rs1351394 | Weight | -0.02545 | 0.002124 | 4.51E-33 | UKBB | FI |

**Table S7**  Continued.

| SNP | Trait | BETA | SE | P | PMID | Exposure |
| --- | --- | --- | --- | --- | --- | --- |
| rs1351394 | Whole body fat mass | -0.01387 | 0.002372 | 5.06E-09 | UKBB | FI |
| rs1351394 | Whole body fat-free mass | -0.02723 | 0.001537 | 3.38E-70 | UKBB | FI |
| rs1351394 | Whole body water mass | -0.02704 | 0.001539 | 4.70E-69 | UKBB | FI |
| rs1474696 | Hip circumference | -0.01435 | 0.002406 | 2.47E-09 | UKBB | FI |
| rs2943646 | Body mass index in males greater than 50 years of age | 0.03 | 0.0051 | 3.30E-09 | 26426971 | FI |
| rs2943646 | Body mass index in males | 0.0237 | 0.0042 | 1.31E-08 | 25673413 | FI |
| rs2943646 | Waist circumference in males | 0.033 | 0.0048 | 5.80E-12 | 25673412 | FI |
| rs2943646 | Body fat percentage | 0.01534 | 0.001948 | 3.41E-15 | UKBB | FI |
| rs3775380 | Body fat percentage | -0.01294 | 0.001868 | 4.34E-12 | UKBB | FI |
| rs3775380 | Hip circumference | -0.01692 | 0.002413 | 2.34E-12 | UKBB | FI |
| rs3775380 | Whole body fat mass | -0.01324 | 0.002377 | 2.54E-08 | UKBB | FI |
| rs459193 | Waist circumference adjusted for BMI | -0.023 | 0.0036 | 1.50E-10 | 25673412 | FI |
| rs459193 | Waist hip ratio | -0.026 | 0.0038 | 6.00E-12 | 25673412 | FI |
| rs459193 | Waist cirumference adjusted for smoking in males | -0.0362 | 0.0056 | 8.85E-11 | 28443625 | FI |
| rs459193 | Waist circumference adjusted for smoking | -0.023 | 0.0042 | 3.32E-08 | 28443625 | FI |
| rs459193 | Body fat percentage | -0.01182 | 0.002136 | 3.11E-08 | UKBB | FI |
| rs6905288 | Waist hip ratio | NA | NA | 4.72E-10 | 20935629 | FI |
| rs6905288 | Waist hip ratio | 0.027 | 0.0037 | 3.10E-13 | 25673412 | FI |
| rs6905288 | Waist hip ratio | 0.024 | 0.0036 | 1.10E-11 | 25673412 | FI |
| rs6905288 | Waist hip ratio | NA | NA | 2.27E-26 | 20935629 | FI |
| rs7012814 | Body mass index | 0.01623 | 0.002423 | 2.11E-11 | UKBB | FI |
| rs7133378 | Waist hip ratio | -0.02 | 0.0035 | 2.20E-08 | 25673412 | FI |
| rs7133378 | Body fat percentage | 0.01911 | 0.002003 | 1.44E-21 | UKBB | FI |
| rs7133378 | Hip circumference | 0.02127 | 0.002587 | 2.03E-16 | UKBB | FI |
| rs7133378 | Whole body fat mass | 0.01775 | 0.002549 | 3.28E-12 | UKBB | FI |
| rs73013411 | Hip circumference | 0.01985 | 0.003573 | 2.80E-08 | UKBB | FI |

**Table S7** Continued.

| SNP | Trait | BETA | SE | P | PMID | Exposure |
| --- | --- | --- | --- | --- | --- | --- |
| rs7903146 | Body mass index | 0.05685 | 0.008601 | 3.85E-11 | 28892062 | FI |
| rs7903146 | Body mass index | 0.0234 | 0.0034 | 1.11E-11 | 25673413 | FI |
| rs7903146 | Body mass index | 0.029 | 0.0025 | 1.90E-31 | 29273807 | FI |
| rs7903146 | Hip circumference | 0.026 | 0.0039 | 2.10E-11 | 25673412 | FI |
| rs7903146 | Hip circumference | 0.027 | 0.0038 | 4.80E-12 | 25673412 | FI |
| rs7903146 | Body mass index | 0.023 | 0.003379 | 1.00E-11 | 25673413 | FI |
| rs7903146 | Body mass index | 0.024 | 0.003366 | 1.00E-12 | 25673413 | FI |
| rs7903146 | Body mass index | 0.029 | 0.004391 | 4.00E-11 | 25673413 | FI |
| rs7903146 | Body mass index | 0.01569 | 0.002643 | 2.93E-09 | UKBB | FI |
| rs7903146 | Hip circumference | 0.02146 | 0.002646 | 5.10E-16 | UKBB | FI |
| rs7903146 | Weight | 0.01288 | 0.002335 | 3.42E-08 | UKBB | FI |
| rs7903146 | Whole body fat mass | 0.01486 | 0.002607 | 1.21E-08 | UKBB | FI |
| rs9884482 | Weight | 0.01265 | 0.002197 | 8.45E-09 | UKBB | FI |
| rs9884482 | Whole body fat-free mass | 0.01301 | 0.00159 | 2.72E-16 | UKBB | FI |
| rs9884482 | Whole body water mass | 0.01299 | 0.001592 | 3.41E-16 | UKBB | FI |

Beta, linear regression coefficient or log odds ratio. SE, standard error. PMID, PubMed identifier.
