# Supplementary material for: Type 1 diabetes, glycemic traits, and risk of dental caries: a Mendelian randomization study
Source: Front Genet. 2023 Oct 10;14:1230113. doi: 10.3389/fgene.2023.1230113 (PMC10597668; doi:10.3389/fgene.2023.1230113)
Supplement: Supplementary file 1 [file DataSheet1.ZIP › Supplementary Table S8.docx]

**Table S8** MR analysis results of four common methods of T1DM and glycemic traits to dental caries.

| Exposure Source | Outcome Source | Outcome | Exposure | Method | nsnp | b | se | p | OR |
| --- | --- | --- | --- | --- | --- | --- | --- | --- | --- |
| PMID: 32005708 | The FinnGen Biobank | Dental caries | T1DM | IVW | 39 | 0.044 | 0.014 | 0.003 | 1.044 |
| PMID: 32005708 | The FinnGen Biobank | Dental caries | T1DM | MR Egger | 39 | 0.056 | 0.022 | 0.014 | 1.058 |
| PMID: 32005708 | The FinnGen Biobank | Dental caries | T1DM | Weighted median | 39 | 0.041 | 0.019 | 0.009 | 1.041 |
| PMID: 32005708 | The FinnGen Biobank | Dental caries | T1DM | Weighted mode | 39 | 0.049 | 0.016 | 0.004 | 1.050 |
| PMID: 34059833 | The FinnGen Biobank | Dental caries | FG | IVW | 66 | -0.075 | 0.142 | 0.597 | 0.928 |
| PMID: 34059833 | The FinnGen Biobank | Dental caries | FG | MR Egger | 66 | -0.015 | 0.257 | 0.953 | 0.985 |

**Table S8** Continued.

| Exposure Source | Outcome Source | Outcome | Exposure | Method | nsnp | b | se | p | OR |
| --- | --- | --- | --- | --- | --- | --- | --- | --- | --- |
| PMID: 34059833 | The FinnGen Biobank | Dental caries | FG | Weighted median | 66 | -0.062 | 0.224 | 0.781 | 0.940 |
| PMID: 34059833 | The FinnGen Biobank | Dental caries | FG | Weighted mode | 66 | -0.053 | 0.206 | 0.796 | 0.948 |
| PMID: 34059833 | The FinnGen Biobank | Dental caries | HbA1c | IVW | 73 | -0.110 | 0.223 | 0.623 | 0.896 |
| PMID: 34059833 | The FinnGen Biobank | Dental caries | HbA1c | MR Egger | 73 | 1.141 | 0.397 | 0.005 | 3.131 |
| PMID: 34059833 | The FinnGen Biobank | Dental caries | HbA1c | Weighted median | 73 | 0.015 | 0.303 | 0.960 | 1.016 |
| PMID: 34059833 | The FinnGen Biobank | Dental caries | HbA1c | Weighted mode | 73 | 0.167 | 0.361 | 0.644 | 1.182 |

**Table S8** Continued.

| Exposure Source | Outcome Source | Outcome | Exposure | Method | nsnp | b | se | p | OR |
| --- | --- | --- | --- | --- | --- | --- | --- | --- | --- |
| PMID: 34059833 | The FinnGen Biobank | Dental caries | FI | IVW | 38 | 0.841 | 0.267 | 0.002 | 2.318 |
| PMID: 34059833 | The FinnGen Biobank | Dental caries | FI | MR Egger | 38 | -0.065 | 0.850 | 0.940 | 0.937 |
| PMID: 34059833 | The FinnGen Biobank | Dental caries | FI | Weighted median | 38 | 0.619 | 0.400 | 0.122 | 1.856 |
| PMID: 34059833 | The FinnGen Biobank | Dental caries | FI | Weighted mode | 38 | 0.428 | 0.588 | 0.472 | 1.534 |
