# Supplementary figures and images for: Type 1 diabetes, glycemic traits, and risk of dental caries: a Mendelian randomization study
Source: Front Genet. 2023 Oct 10;14:1230113. doi: 10.3389/fgene.2023.1230113 (PMC10597668; doi:10.3389/fgene.2023.1230113)

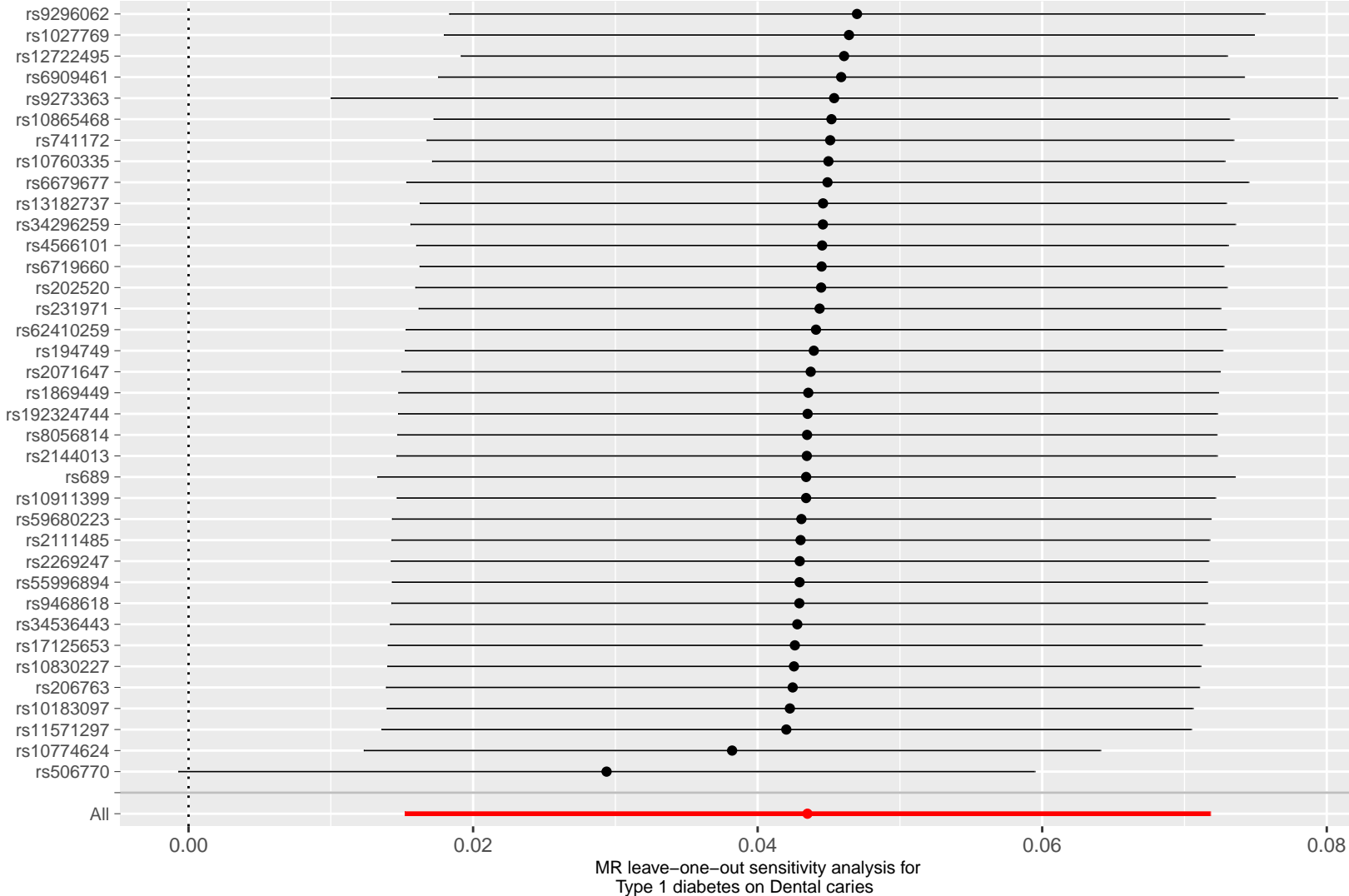

Supplement: Supplementary file 1 [file DataSheet1.ZIP › Supplementary Figure S1.pdf]

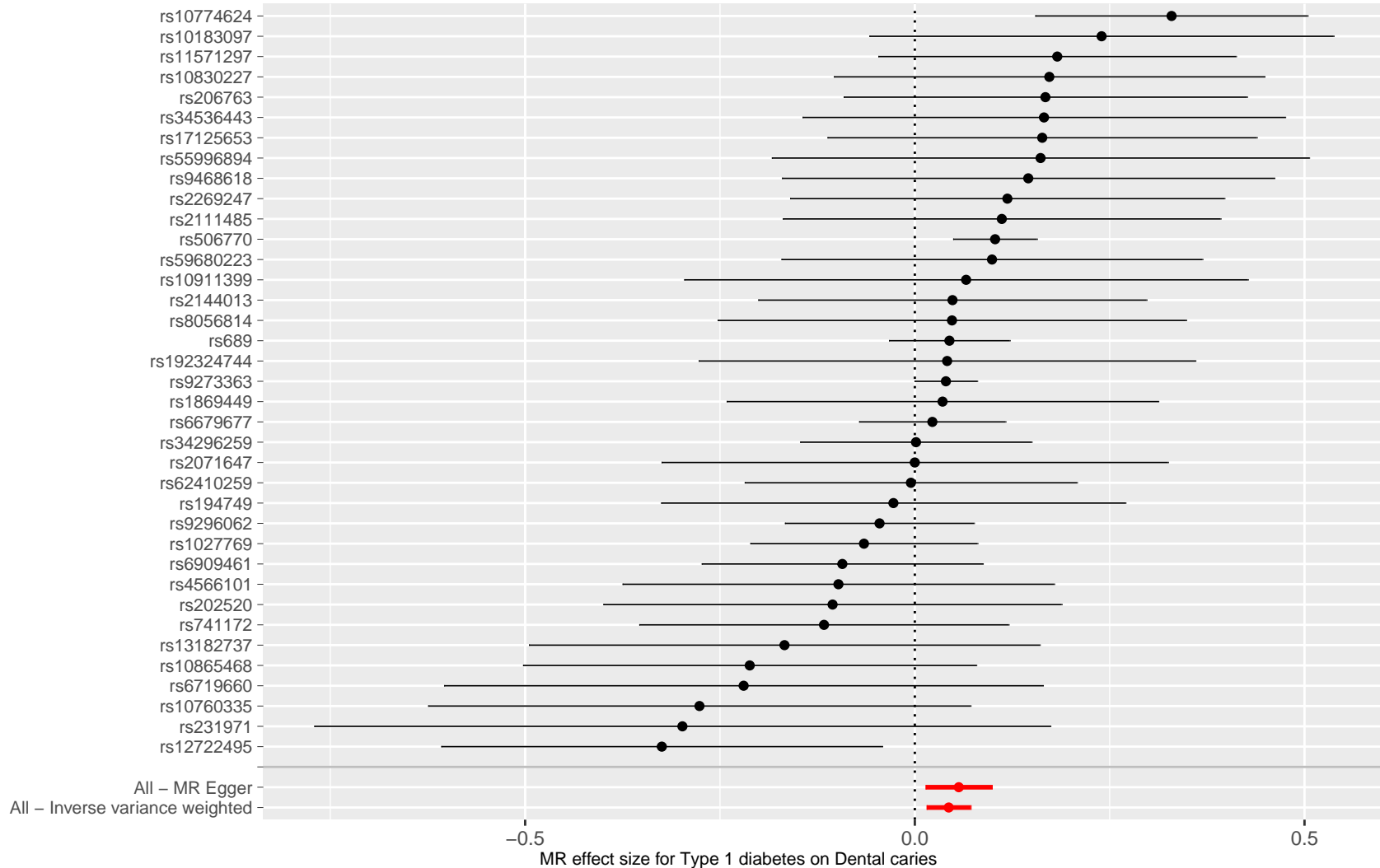

Supplement: Supplementary file 1 [file DataSheet1.ZIP › Supplementary Figure S2.pdf]

# MR Method

- Inverse variance weighted
- MR Egger

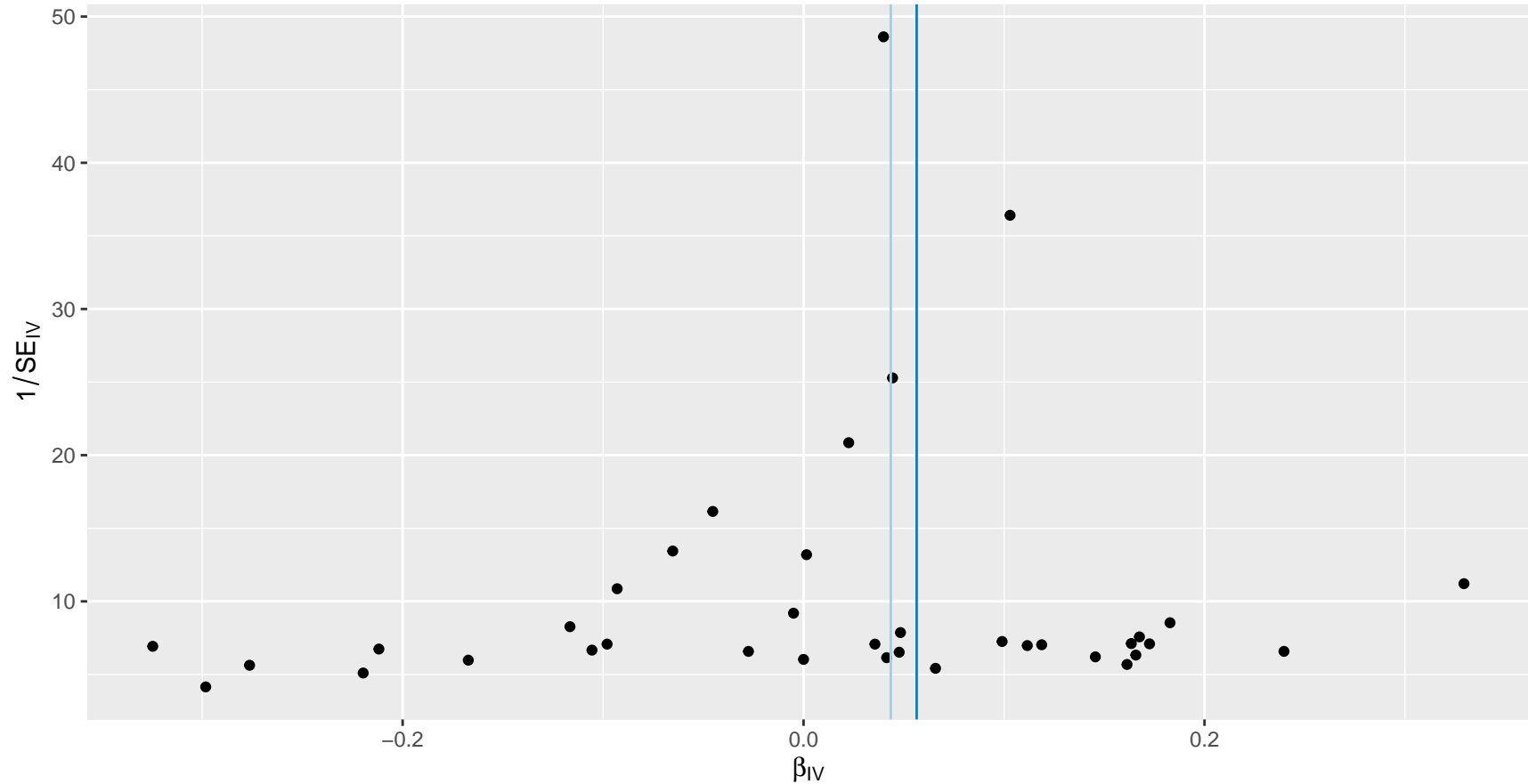

Supplement: Supplementary file 1 [file DataSheet1.ZIP › Supplementary Figure S3.pdf]

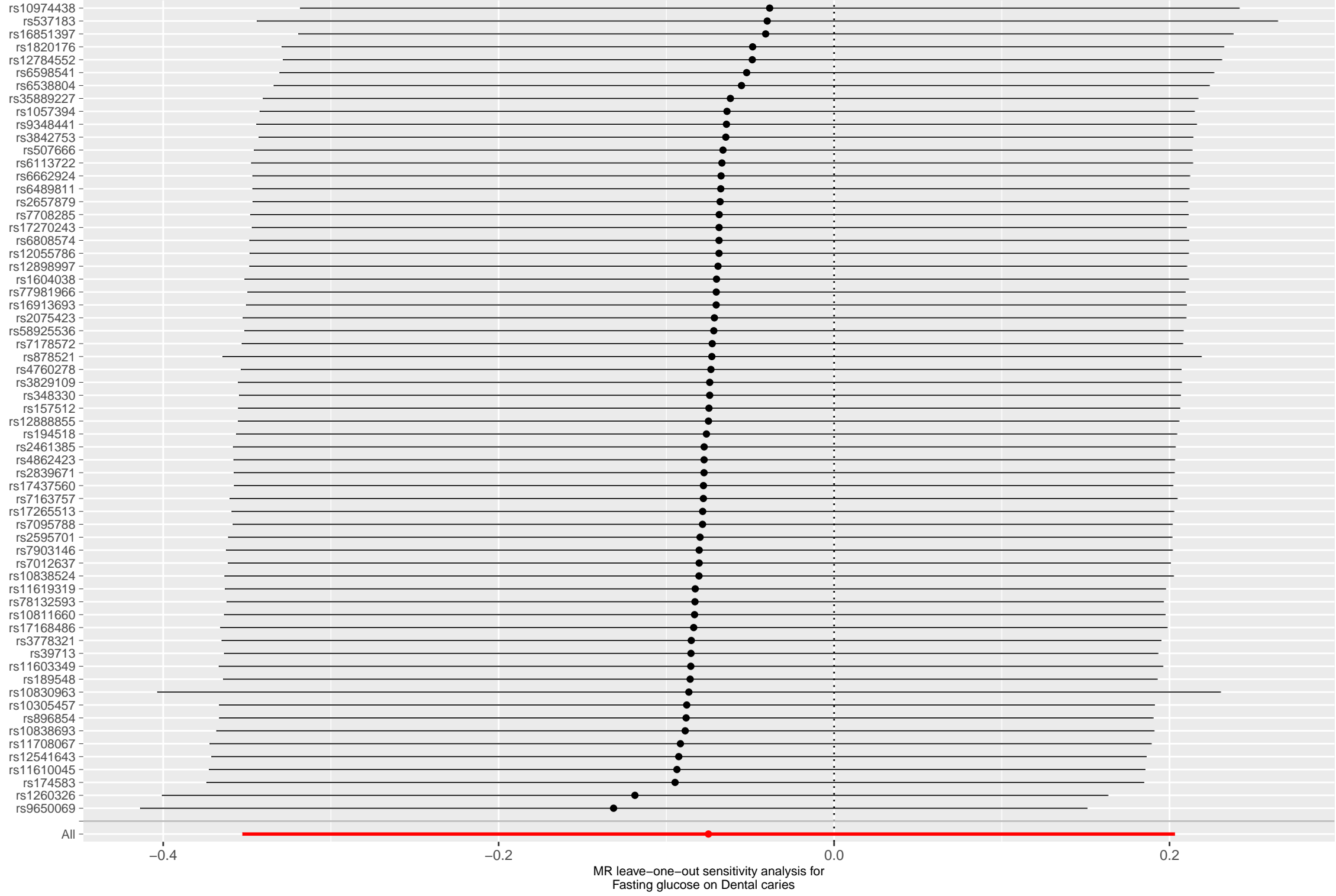

Supplement: Supplementary file 1 [file DataSheet1.ZIP › Supplementary Figure S4.pdf]

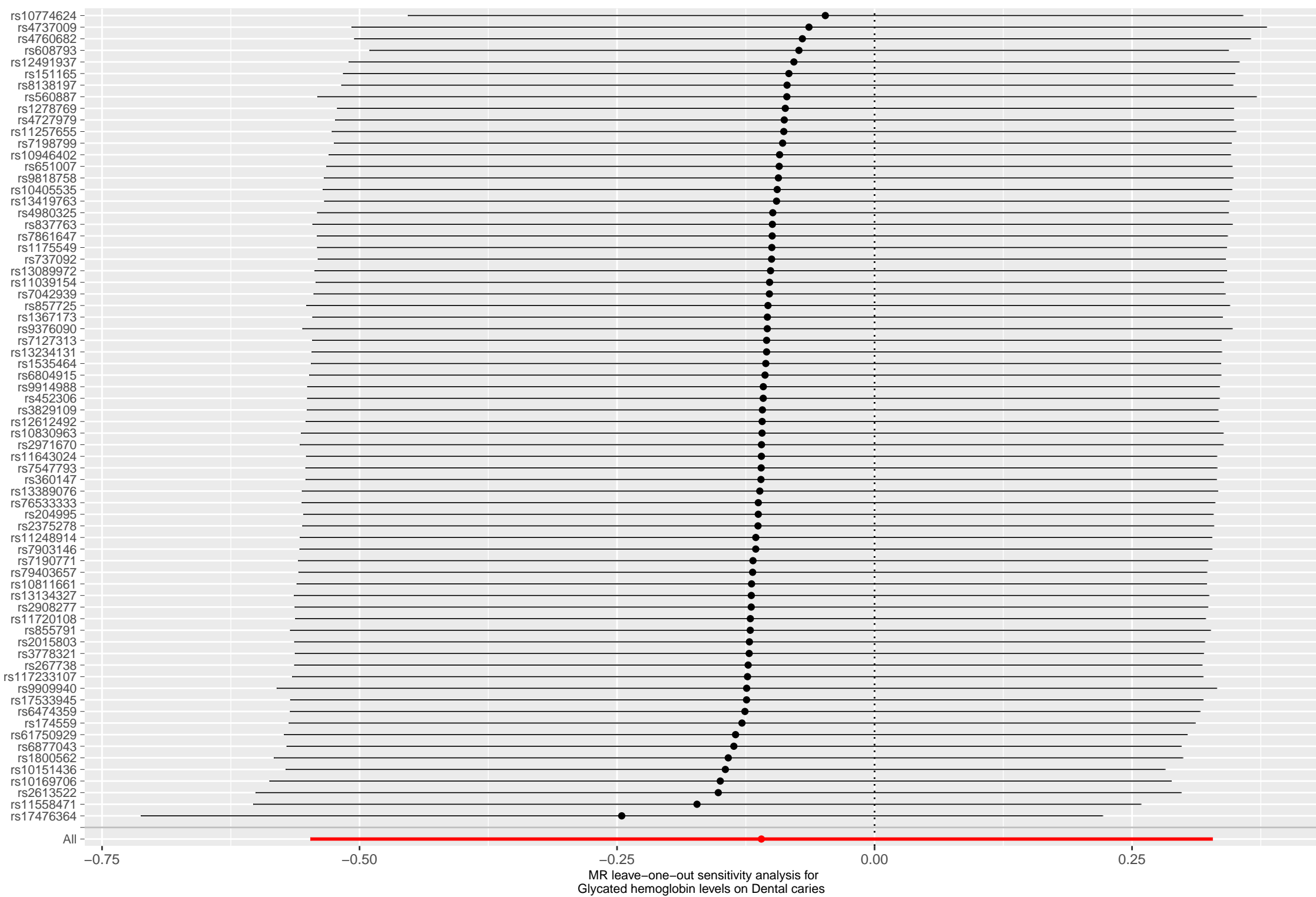

Supplement: Supplementary file 1 [file DataSheet1.ZIP › Supplementary Figure S5.pdf]

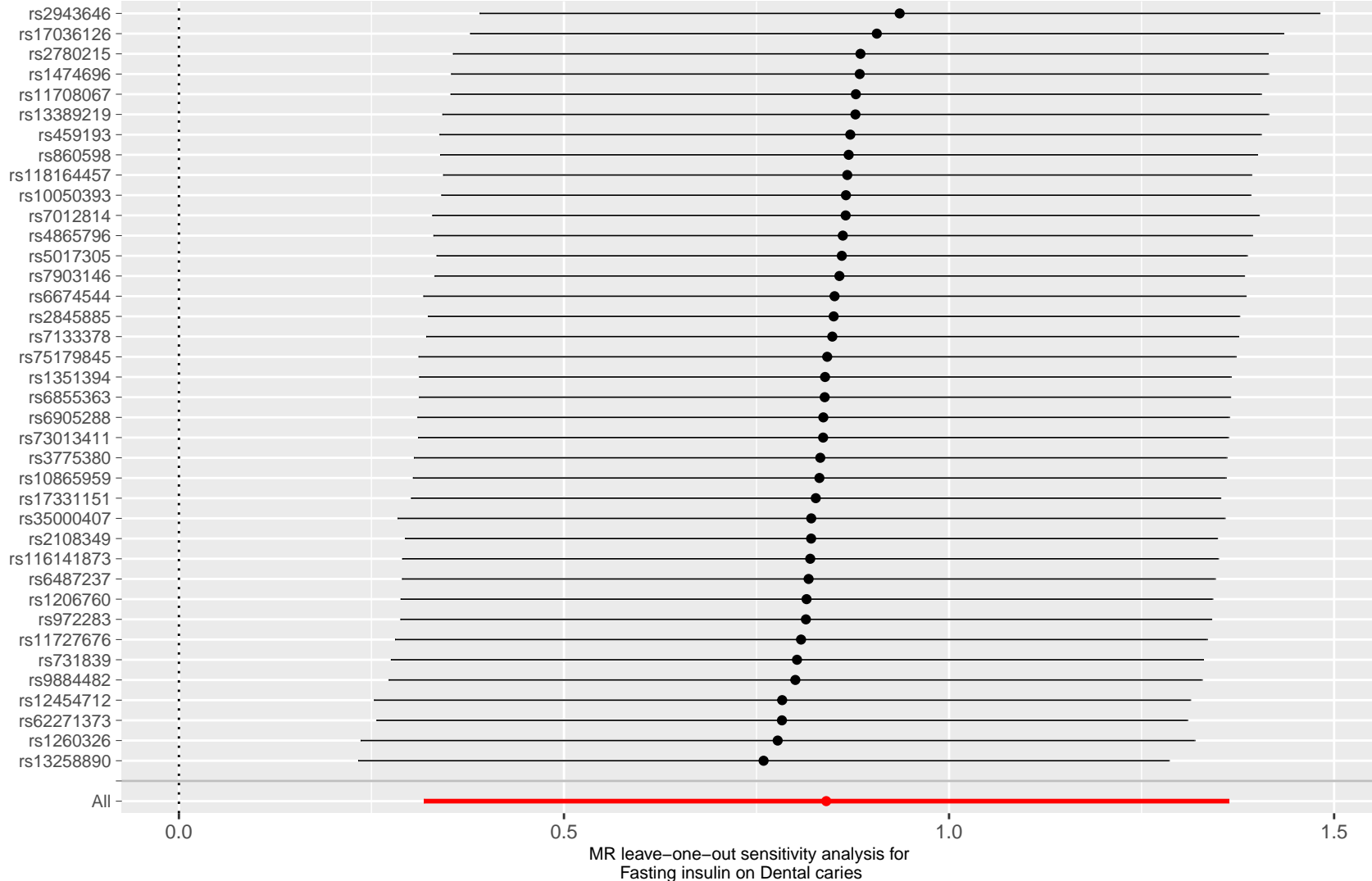

Supplement: Supplementary file 1 [file DataSheet1.ZIP › Supplementary Figure S6.pdf]
